# Design and formative usability evaluation of a prototype integrated digital health platform for chronic care support

**DOI:** 10.3389/fdgth.2026.1780895

**Published:** 2026-10-22

**Authors:** Kekayan Nanthakumar, Kunal Gupta, Alireza F. Nia, Sergio Dempsey, Nastaran Saffaryazdi, Alaeddin Nassani, Elizabeth Broadbent, Merryn Tawhai, Gonzalo Maso Talou

**Affiliations:** 1 Auckland Bioengineering Institute, The University of Auckland, Auckland, New Zealand; 2 Department of Computer Science and Engineering, University of Aizu, Aizuwakamatsu, Japan; 3 Department of Psychological Medicine, Faculty of Medical and Health Sciences, Psychological Medicine, The University of Auckland, Auckland, New Zealand

**Keywords:** chronic care management, chronic disease management, conversational agents, digital health, embodied conversational agent, health coaching, large language models

## Abstract

**Introduction:**

Chronic care management platforms often support only one or two core pillars of care, such as patient monitoring, patient activation, and data-driven care coordination. This fragmentation can limit the continuity of support and reduce the usefulness of shared patient data among patients, coaches, and clinicians.

**Method:**

This study describes the design, software architecture, and formative usability evaluation of the Digital Health Navigator (DiNa) platform, an integrated system designed to bring together the core pillars of chronic care management. DiNa combines wearable and self-reported monitoring, a large language model-powered embodied conversational agent, and a coordinator dashboard within a single modular ecosystem. DiNa was implemented as an Android application, a web-based coordinator dashboard, a microservices backend, and wearable integrations with a smartwatch and a continuous glucose monitor. The embodied conversational agent supports goal setting, logging, reminders, and knowledge-grounded education through coaching informed by motivational interviewing and cognitive behavioural therapy. We conducted moderated laboratory think-aloud usability sessions with 10 relatively young healthy volunteers familiar with digital health tracking. Participants explored the application and completed structured tasks, including setting goals, logging meals with an image, reviewing history, interacting with the coach, and viewing trends.

**Results:**

Outcomes included task performance, the System Usability Scale (SUS), a brief NASA Task Load Index (NASA-TLX), three acceptability items, and a reflexive thematic analysis of interviews. Participants completed the core tasks in a facilitated session, and a small number of critical breakdowns were observed and quantified. Mean SUS was 76.1 (SD 15.0), indicating generally good usability. Overall, the NASA-TLX workload averaged 26.7, with mental demand and effort higher than the other subscales and the remaining subscales low. Acceptability ratings indicated moderate early acceptability, with perceived usefulness rated more positively than near-term use or recommendation. Qualitative analysis identified five themes, including burdensome and sometimes unclear logging workflows, uneven goal setting, a friendly but occasionally generic coach, interface elements that reduced confidence, and a strong interest in integration with existing devices and applications.

**Discussion:**

These findings provide early evidence that an integrated platform combining continuous sensing, conversational coaching, and clinician-facing coordination is technically feasible and usable in a controlled setting with healthy volunteers, and they identify the workflow refinements needed before evaluation in clinically representative populations.

## 1 Introduction

Chronic diseases, including heart disease, diabetes, cancer, stroke, and dementia represent the dominant global health challenge of the twenty-first century, accounting for an estimated 63% of worldwide deaths and imposing an unprecedented economic burden projected to reach $47 trillion by 2030 (1, 2). Part of this burden is fundamentally rooted in modifiable lifestyle and behavioural risk factors: physical inactivity, poor nutrition, tobacco use, and excessive alcohol consumption. These collectively account for more than 50% of preventable disease deaths (2). Comorbidities compound this challenge, with multiple concurrent chronic conditions necessitating complex coordinated care over long periods (3).

Technological advances in both hardware and software, in conjunction with a global imperative to reduce clinical service costs and improve patient outcomes, have catalysed the development of a diverse range of digital platforms for chronic care management (CCM) at home (4). Chronic diseases generally persist over an extended period and progress slowly, affecting the quality of life of individuals and necessitating long-term monitoring and management (5). CCM platforms that enable continuous health monitoring, patient engagement, and coordinated care planning have demonstrated a reduction in the economic and logistical burdens of chronic care for providers, clinicians, families, and patients alike (6, 7). The effectiveness of these platforms is based on three interconnected pillars (detailed in Section 2): (i) *Patient Monitoring*, (ii) *Patient Activation*, and (iii) *Data-Driven Care Coordination* (5).

Although each of these pillars is well represented in prior research, many existing platforms emphasise only one or two of them and therefore provide only partial support for chronic care management (8, 9). For example, platforms such as MySugr focus primarily on patient monitoring through glucose tracking and logging, with more limited behavioural support features (10). By contrast, behavioural intervention apps such as Noom emphasise patient activation through coaching but do not provide comprehensive physiological monitoring (11). Care coordination platforms such as Epic MyChart support provider communication but typically do not incorporate real-time monitoring or personalised behavioural support (12). As a result, patients may need to use multiple tools to meet different chronic care needs, which can increase the burden and reduce continuity across self-management activities (9).

Beyond the practical inconvenience of using multiple applications, fragmentation can disrupt care processes that depend on a shared context. When monitoring and coaching are separated, patient-generated signals, such as post-meal glucose excursions, missed medication doses, symptoms, or meal logs, may not readily inform context-sensitive support. Similarly, when clinicians review monitoring data without visibility into recent goals, coaching history, or self-reported context captured elsewhere, care planning may rely only on a partial view of the patient’s status. Fragmentation in chronic disease care has also been associated with increased emergency department visits, duplicated testing, and higher healthcare costs (9). These limitations point to a system-level gap: *current CCM platforms lack tightly coupled feedback loops in which monitoring data, behavioural support, and clinical review operate over a shared and continuously updated context.* Integrated systems have the potential to address this gap by enabling closed-loop interactions, where patient-generated data informs coaching in real time, and both are available for retrospective clinical decision-making. However, it remains unclear how such integration affects user interaction, usability, and perceived value in practice.

To address this, we present the Digital Health Navigator (DiNa), a prototype digital health platform designed to operationalise shared-context interactions across the three pillars. DiNa offers passive and active remote patient monitoring and integration of wearable sensors through a mobile application. To support patient activation, DiNa uses a tool-based conversational agent that interprets user context, manages goals and action plans, and invokes domain-specific tools such as meal logging, reminders, and knowledge-based guidance. This interaction is delivered through a digital human interface, enabling conversational, context-aware behavioural coaching grounded in user data. These components are coordinated through a backend system and clinician-facing dashboard to support data-driven care coordination within a unified architecture. This work focuses on the interaction implications of such integration. Specifically, we investigate whether an integrated CCM workflow can achieve acceptable usability, cognitive workload, and user-perceived value in a controlled setting, and what interaction challenges emerge when monitoring, coaching, and coordination are combined within a single system.

In Section 3, we present the design and architecture of the system. Section 4 describes the formative usability study, followed by the results in Section 5 and the discussion of implications and limitations.

## 2 Chronic care management digital platforms overview

### 2.1 Pillar 1: patient monitoring

Patient monitoring focuses on collecting health data to track patient status, enhanced by remote measurement technologies (RMTs), such as smartphones, wearables, and associated applications, to collect data on human behaviour, psychological states, and physiological parameters (13, 14). RMTs commonly use two complementary approaches to data collection. Active RMTs require users to input data, including subjective measures such as mood and pain, and behavioural data such as medication adherence and dietary intake (15–17). This self-reported data is then often synchronised with clinical dashboards to support timely intervention (18) and remains essential to understanding self-care behaviours in chronic disease management (19). For example, in diabetes management, self-reported information such as medication adherence, hypoglycemia symptoms, meal context, and food intake diary can complement sensor-derived data by providing behavioural and experiential context not directly captured by physiological monitoring alone (20).

Passive RMTs, in contrast, collect data through embedded sensors in devices such as wrist-worn trackers, patch monitors, and smart scales, providing continuous measurements of physiological and activity-related signals (21–25). When integrated into digital platforms, these data streams enable longitudinal tracking and have been associated with improved self-management and earlier detection of clinical risks (26, 27).

Despite these advances, existing monitoring systems primarily focus on data collection and visualisation, often operating independently of behavioural support and clinical coordination processes (4, 9). For instance, platforms such as Glooko aggregate data from multiple devices into clinician dashboards (28), but do not directly support behavioural intervention. Conversely, research systems such as PhysioLLM (29) and the Personal Health Insights Agent (30) demonstrate that conversational agents can interpret wearable data, yet do not integrate these insights into clinician-facing workflows.

As a result, patient-generated data is often not translated into timely, context-aware action. Monitoring systems can capture rich physiological and behavioural signals, but without integration into coaching and care coordination, these data remain underutilised, limiting their role in driving meaningful and responsive interventions throughout the care continuum.

### 2.2 Pillar 2: patient activation

Patient activation encompasses the knowledge, confidence, and ability of individuals to manage their health within a CCM platform. Supporting sustained behaviour change requires approaches that combine education, engagement, and adherence strategies (8, 31). To this end, CCM platforms incorporate evidence-based behaviour change techniques (BCTs), such as goal setting, real-time feedback, and motivational reminders (32, 33), alongside health education resources and interactive modalities, including secure messaging, telemedicine, and embodied conversational agents (ECAs) (34, 35). These approaches aim to promote proactive self-management and improve adherence to treatment and lifestyle recommendations (36).

Despite these advances, sustaining engagement remains a major challenge. Many digital health interventions experience rapid attrition, and studies report that up to 80% of users disengage within the first week in self-guided settings (37, 38). To address this, systems increasingly incorporate strategies such as gamification, personalised nudges, and just-in-time adaptive interventions that aim to provide support based on user behaviour (39–44). In particular, AI-powered ECAs have shown promise in improving engagement by enabling on-demand conversational support, and users often report higher satisfaction and comfort compared to traditional interfaces (18, 45–49).

However, many activation systems operate with limited access to the physiological and behavioural context of users in real-time. As a result, behavioural support is often provided in a generic or reactive manner, rather than dynamically tailored to the current state of the user. Furthermore, while many systems incorporate elements of behaviour change techniques, these are not always tightly integrated with user data streams or interaction history, limiting their ability to provide coherent, personalised, and adaptive coaching over time. Consequently, although activation systems can deliver structured behavioural support, their effectiveness is constrained by a lack of context-aware integration with monitoring and care coordination, reducing their ability to provide timely, relevant, and actionable guidance in real-world use.

### 2.3 Pillar 3: data-driven care coordination

Data-driven care coordination supports personalised and timely decision-making by integrating patient data with collaborative workflows across multidisciplinary healthcare teams (50, 51). Contemporary CCM platforms incorporate functionalities such as risk stratification, clinical alerts, and dynamic care plan updates (52), alongside communication tools including secure messaging, shared dashboards, and telehealth consultations to improve continuity of care (53, 54). Interoperability standards such as Fast Healthcare Interoperability Resources (FHIR) (55) further enable structured data exchange across distributed healthcare systems (56).

Recent advances in large language models (LLMs) have extended these capabilities by enabling more flexible interpretation of patient data and supporting clinician-facing insights. For example, systems such as PhysioLLM (29) and the Personal Health Insights Agent (30) demonstrate how LLMs can synthesise wearable data and provide personalised explanations or recommendations. These developments suggest a shift towards more adaptive and data-driven coordination workflows.

However, existing care coordination systems remain primarily clinician-facing and are often loosely coupled with patient-facing monitoring and behavioural support tools. As a result, care coordination systems are often limited to aggregated physiological data, with limited access to the behavioural, contextual, or interactional processes that underlie these signals. For example, clinicians may observe trends in glucose or activity without visibility into recent goals, coaching interactions, or patient-reported context that could help interpret these patterns. Consequently, although care coordination platforms support data-driven workflows, they are constrained by incomplete context. Without integration across monitoring and activation systems, coordination remains largely retrospective, limiting the potential for more personalised and responsive care.

### 2.4 Aims

Collectively, these limitations suggest a system-level gap in current chronic care management platforms: monitoring, behavioural support, and care coordination are often available but not well connected through shared context. DiNa was therefore designed as a modular platform that connects monitoring, behaviour-informed conversational coaching, and clinician-facing review within a shared data architecture.

This study provides a formative evaluation of the interaction implications of this integration. The evaluation uses relatively young, healthy adults familiar with digital health tracking as surrogate users, following a staged HCI evaluation strategy in which interaction and pipeline defects are identified before testing with the intended clinical population (57). It therefore examines interaction feasibility and informs design refinement, with clinical effectiveness, long-term engagement, and adoption among people with chronic conditions deferred to future clinical evaluation.

Accordingly, the study addresses two overarching questions, which Section 4 operationalises as four research questions (RQ1 to RQ4):

1. What are the usability, cognitive workload, and perceived acceptability of the integrated DiNa platform, as assessed through standardised measures (SUS, NASA-TLX) and self-reported ratings?
2. What usability barriers and workflow breakdowns emerge during core task completion, and what design implications do they suggest for future system refinement and evaluation?

To contextualise these questions, the paper first presents the design and technical implementation of the DiNa platform (Section 3).

## 3 DiNa platform

### 3.1 Platform design

Our DiNa platform addresses the challenge of fragmentation in chronic care management through the unified ecosystem built on four key components:

1. A smartphone application to engage users and collect available health data.
2. A web-based coordinator dashboard for clinician or research summary reporting and closing the care loop through remote monitoring and clinical decision support.
3. A backend system for handling data storage, processing and integrations.
4. The wearable integration enabling continuous health monitoring.

All three pillars are vertically represented across these components. The decoupling of smartphone application (patient user), dashboards (specialist/carer user), storage and processing (bioengineering engine) and sensors, gives the platform versatility to adapt to different applications where one or many of these components need to be modified to satisfy the demands of a specific condition or strategy of care.

Additional technical details of platform components are provided in the Supplementary Material.

### 3.2 Smartphone application

The DiNa smartphone application serves as the primary user-facing interface, offering personalised digital health coaching and interactive features to empower users in their daily health management. Key features and panels are shown in Figure 1. Central to this interaction is DiNa , a realistic rendering of a digital human companion implemented via Soul Machines Studio^1^ integrated with an AI agent (Section 3.3). DiNa engages users through natural conversations in both voice and text, providing personalised behavioural coaching and numerous supportive interactions.

**Figure 1:**
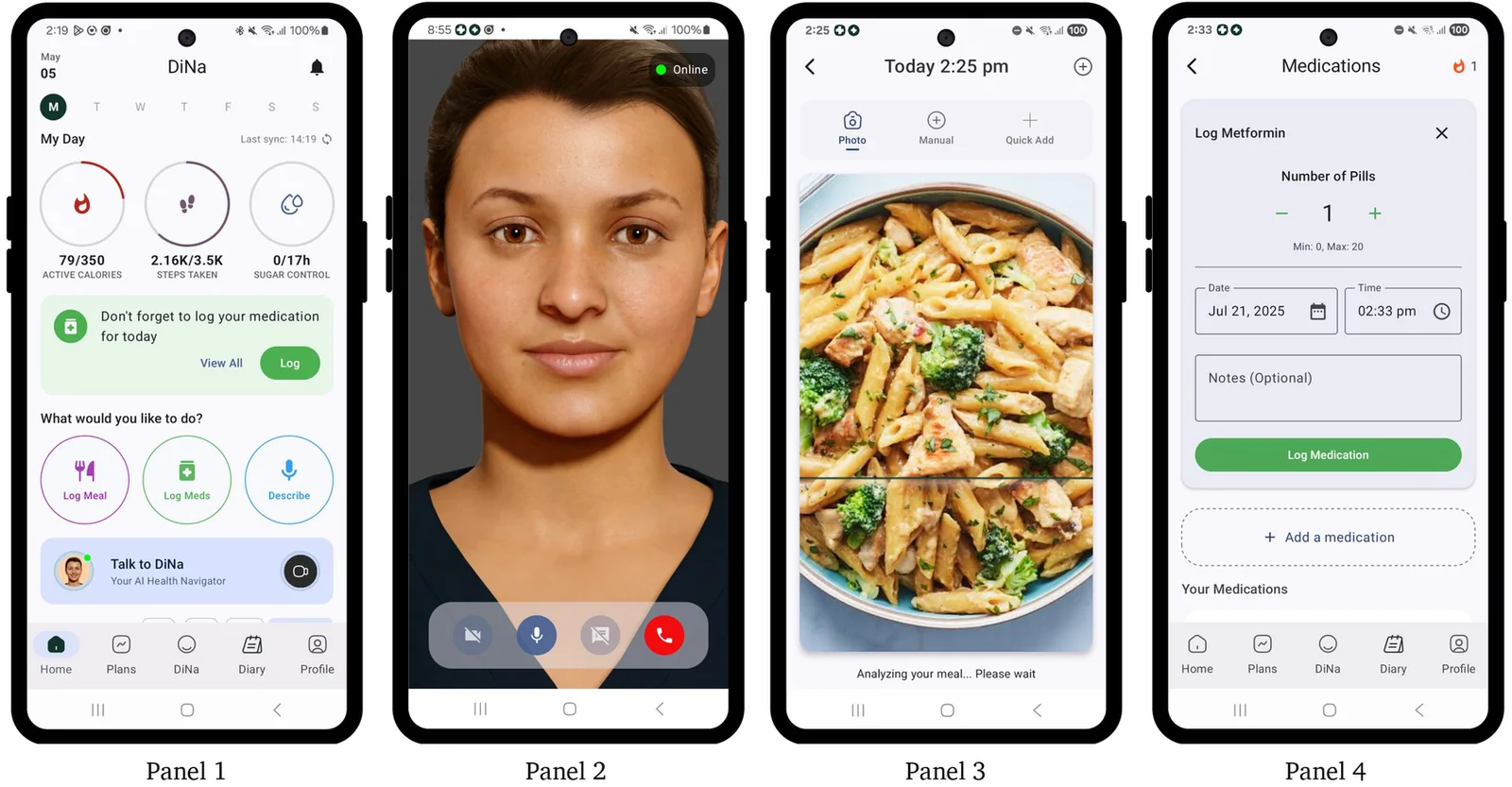
Key panels of the smartphone application. From left to right: [Panel **(1)**] The home dashboard, where available health data (e.g., active calories, sugar control, etc) are portrayed. (Panel **(2)**] The embodied conversational digital human agent DiNa informs users about health information, goal setting, and logging information. [Panel **(3)**] Meal logging panel with automatic food identification. [Panel **(4)**] Medication logging.

The smartphone application includes features for setting and managing health goals, tracking medication adherence, recording meals and symptoms, and receiving reminders related to goals and medications. It also presents continuous health data from connected devices such as heart rate, glucose trends, and activity data. These data are used to support coaching interactions and provide context for goal setting, reflection, and self-management.

Data collection within the application supports multiple user-generated inputs, including meal logs, medication intake, and self-reported symptoms. These data are stored locally before synchronisation with the cloud backend. Once synchronised, they become available for coaching, longitudinal review, and clinician-facing coordination. The offline-first design allows the application to continue functioning in the absence of network connectivity, with local caching used until synchronisation is restored or the allocated storage threshold is reached. The application was implemented as a native Android application in Kotlin.

### 3.3 Conversational coaching agent

The embodied conversational agent (ECA) is the core interaction component. Presentation and dialogue are separated. Soul Machines Studio renders the digital human and handles audiovisual delivery, and a separate tool-calling coaching agent generates content and invokes platform actions (Figure 2). The coaching LLM is Claude Sonnet 4.5 at temperature 0. Complex queries are delegated to a reasoning sub-agent running Claude Opus 4.6, which returns a structured recommendation to the coaching agent and never addresses the user, so a single agent remains accountable for every user-facing utterance.

**Figure 2:**
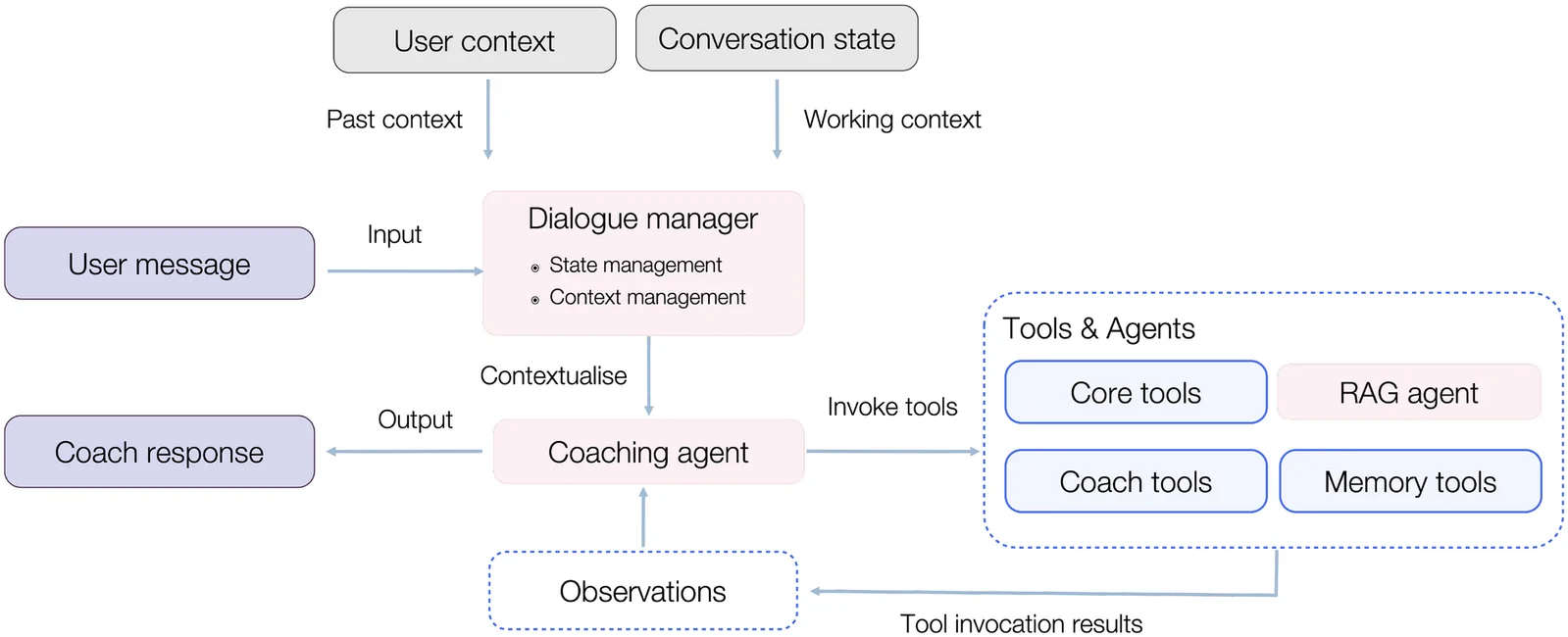
Overview of the conversational flow and the structure of the conversational agent. The dialogue manager processes the user message and maintains conversation state and user context. The coaching agent then generates a response directly or uses external modules, including retrieval, core tools, coach tools, and memory tools, before returning the final response.

The dialogue manager owns the session lifecycle. It validates input, persists the user message, assembles context, and invokes the agent loop. At session start, a context snapshot of the user’s available records (goals, recent logs, wearable summaries, and memory entries) is appended after the system prompt so that coaching is grounded in the user’s current state from the first turn.

#### 3.3.1 Agent loop

The coaching agent runs an agentic tool loop. At each iteration, it returns either a user-facing message or one or more tool calls. Tool results are appended to the working context and the loop repeats until a user-facing message is produced or a bounded iteration limit is reached. Independent tools may execute concurrently. Every tool call and result is persisted as an auditable record, which gives the coordinator dashboard and later analysis a complete trace of the actions taken on the user’s behalf. Tools fall into three groups, namely shared self-management tools (meal and medication logging, goals, reminders, historical data retrieval), diabetes-specific tools, and memory tools. This tool-based design lets the agent combine dialogue with concrete platform actions and prevents it from operating as a text generator alone.

#### 3.3.2 Coaching protocol

The coaching protocol is implemented through structured tool calls within the agent loop. During exploratory coaching, the agent uses MI-informed strategies to explore motivation, ambivalence, and readiness. When a specific behavioural barrier is identified, it may select BCT or CBT-informed techniques for problem solving, coping support, or planning. At each coaching decision point, the LLM invokes a technique-selection tool that records the selected approach and technique and returns strategy guidance to the agent. When the user is ready to act, the agent may invoke a separate action tool. Agreed actions are retained for later review. The system uses CBT-informed behavioural strategies and does not provide formal psychotherapy. BCTs, named here following BCT Taxonomy v1 (58), are incorporated at two levels that share one data layer. At the platform level, they are built into application mechanisms, and at the conversational level, the coach selects techniques through the technique-selection tool and can execute them through its action tools (Table 1). The techniques delivered in the usability sessions were coded from the transcripts (Section 4.4). Formal fidelity to MI and CBT-informed techniques was not evaluated in this formative study.

**Table 1:** Incorporation of behaviour change techniques in DiNa, mapped to BCT Taxonomy v1 (58).

Level	Mechanism	BCTTv1 technique(s)
Platform	Goal creation with “Make it SMART” and schedule	1.1 Goal setting (behaviour); 1.4 Action planning
Platform	Goal and medication reminders	7.1 Prompts/cues
Platform	Meal, medication, and symptom logging; diary	2.3 Self-monitoring of behaviour
Platform	Nutrient estimates, home summary, glucose and heart-rate trends	2.2 Feedback on behaviour; 2.6 Biofeedback
Coach	Retrieval-grounded education with named sources	4.1 Instruction on how to perform the behaviour; 5.1 Information about health consequences; 9.1 Credible source
Coach	Coaching dialogue with goal, reminder, and logging action tools on the shared data	1.1 Goal setting (behaviour); 1.4 Action planning; 1.5 Review behaviour goal(s); 2.2 Feedback on behaviour; 2.3 Self-monitoring of behaviour; 8.2 Behaviour substitution

#### 3.3.3 Retrieval

Educational answers are grounded by retrieval-augmented generation (RAG) against a curated knowledge base restricted to trusted sources (59). The corpus comprises 147 topics from Healthify and Diabetes New Zealand, the two principal consumer health information providers in New Zealand, and covers T2D, medications including insulin, glycaemic emergencies, cardiovascular comorbidity, nutrition, physical activity, and related lifestyle topics. Documents are split by recursive character chunking and embedded with OpenAI text-embedding-3-small. The top three retrieved chunks are returned with their source URLs, and the user-facing message tool carries a sources field, so that every health claim presented to the user remains attributable to a named page.

#### 3.3.4 Memory

Memory has three layers. Working memory holds the current session and user context. Longer-term per-user memory stores durable facts, communication preferences, and importance-weighted episodic entries, which are updated through dedicated tools and re-injected at the start of the next session. A recall tool provides semantic search over past coaching sessions for questions that refer to earlier conversations.

#### 3.3.5 Guardrails

Guardrails operate across the interaction and action pipeline. Incoming user messages are first screened by a lightweight Llama Guard 4 safety-classification model to identify unsafe or out-of-scope requests before they reach the coaching agent. Prompted scope further prohibits recommending or adjusting medication or insulin, requires escalation for severe glycaemia, emergencies, self-harm, and other out-of-scope situations, and requires health claims to be grounded in tool data or cited sources. Generated responses are screened by the Llama Guard 4 safety classifier before delivery to the user (60). Structural validation applies strict tool schemas and closed enumerations to behavioural fields, preventing the agent from recording values outside the defined framework.

### 3.4 Web-based coordinator dashboard

The web-based coordinator dashboard provides an interface for user monitoring (see Figure 3). The dashboard can be used by healthcare professionals, researchers, or health coaches to assess participants remotely and intervene when necessary.

**Figure 3:**
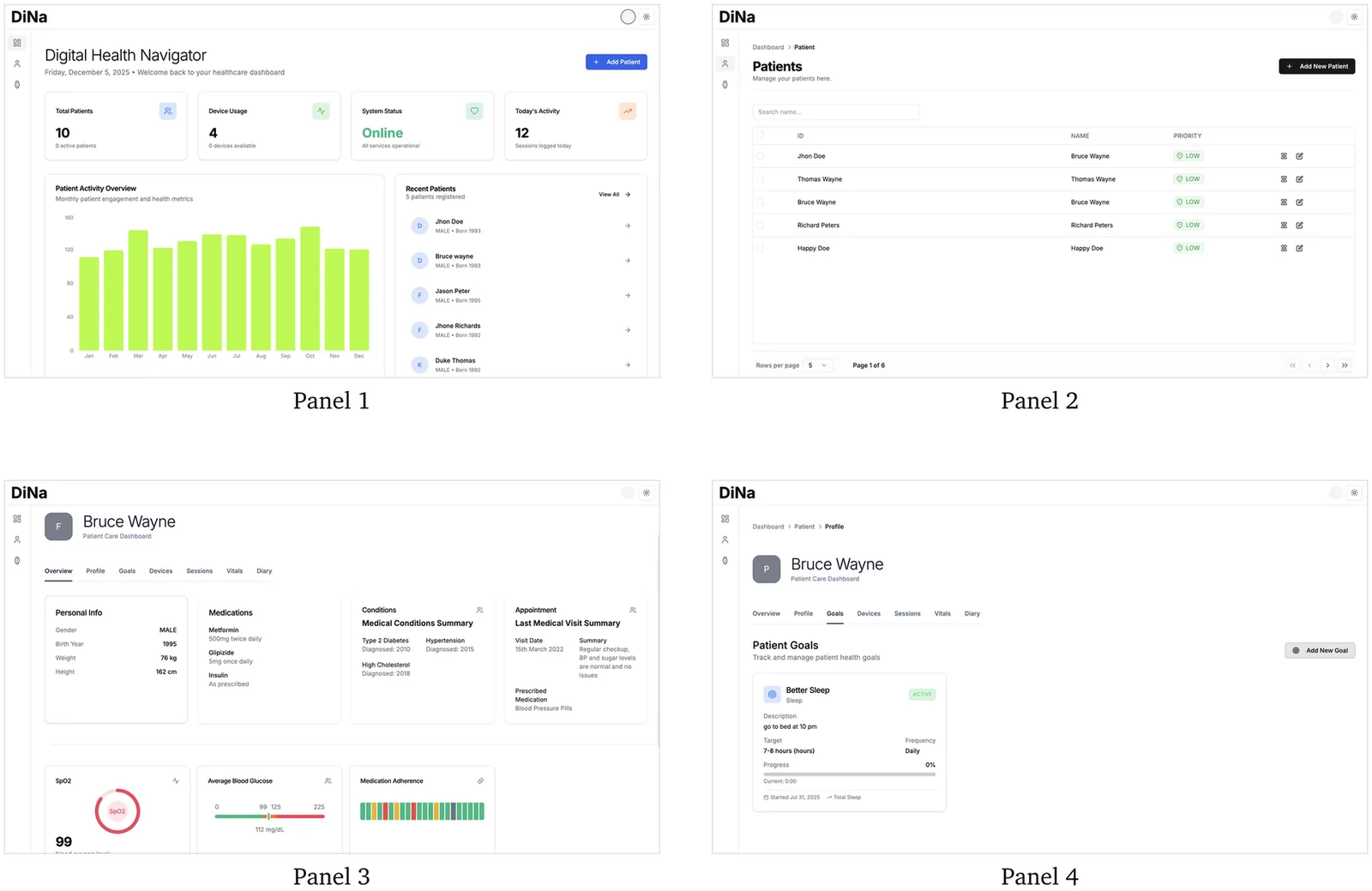
Key panels of the coordinator dashboard. From left to right, top to bottom: [Panel (1)] Home page. [Panel (2)—multi-patient level]. Enrolled users **(fictionalized)** are shown, with priority levels. [Panel (3) and (4)—patient-specific level] Within each patient profile **(fictionalized)**, full details are available to be assessed as needed to personalise communication, treatment, feedback, and adjusted goal setting.

There are two levels of information within the dashboard. The first is a multi-patient reporting screen with priority indicators identifying users who may need assessment. This heat level is based on predefined monitor-user health goals and status. The second patient-specific level provides a detailed view of the patient. This structure supports rapid assessment and more personalised follow-up based on available data.

In addition, this dashboard includes essential administrative features for managing wearable device allocation, tracking platform engagement, and monitoring coaching within the system for operational transparency and oversight of research protocols.

### 3.5 Backend system

CCM platforms must support diverse user needs, high data volumes, and evolving feature sets without compromising reliability or responsiveness. To meet these requirements, the backend of DiNa was designed as a modular service architecture (Figure 4) that supports three platform functions: conversational coaching, multimodal data ingestion, and access to patient data across patient and clinician-facing interfaces.

**Figure 4:**
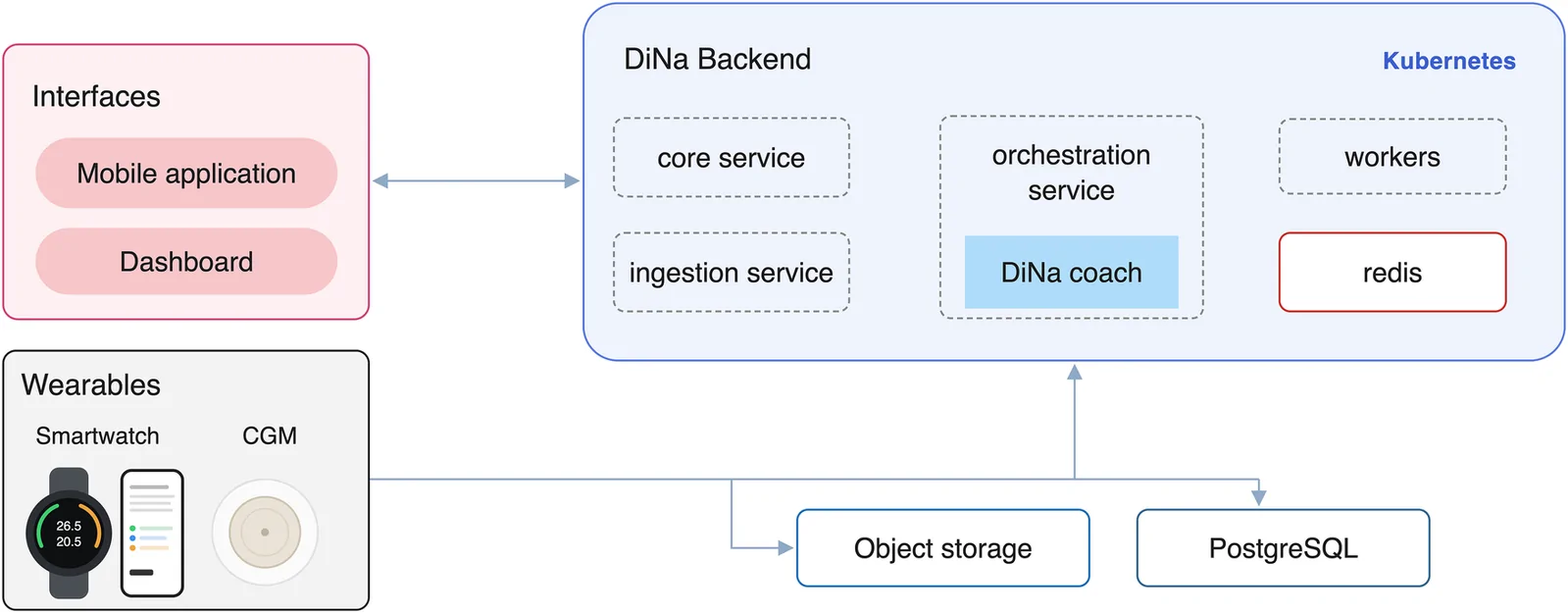
Overview of the DiNa architecture. Patient-facing and clinician-facing interfaces communicate with the DiNa backend. The core service supports application functions, the ingestion service handles incoming device and user data, and the orchestration service hosts the DiNa coach. Worker processes support background tasks. Redis supports caching, transient state, and session coordination for horizontal scaling of the WebSocket-based orchestration service. Services are containerised and deployed on Kubernetes. Wearable and application data are stored in object and relational storage and accessed by backend services as needed.

As shown in Figure 4, the backend comprises four main functional components: an orchestration service, a data service, an ingestion service, and asynchronous worker services. Together, these components manage interactive coaching and logging sessions, persistent storage, cloud-based uploads, background processing, and synchronisation across the mobile application, conversational agent, and coordinator dashboard.

The core of the backend is the orchestration service, which coordinates interactions between the DiNa mobile application, the embodied conversational agent powered by an LLM, and the platform data layer. The agent can be delivered through either a digital avatar or a standard voice or chat interface. During each session, the orchestration service retrieves user context, including physiological measurements, medical background, lifestyle logs, and prior interactions, to support context-aware coaching. Session histories are retained to preserve continuity across repeated engagements and to support later analysis of user engagement and conversational patterns. Interactive coaching, logging, and in-session wearable streaming use WebSocket transport, chosen for low-latency bidirectional communication. Redis provides caching, publish-subscribe messaging, and session coordination, which is the mechanism by which the WebSocket orchestration service scales horizontally across replicas.

The data service provides the persistent data layer for the platform. It maintains user records, device registrations, biosignal data, lifestyle logs, action plans, treatment-related information, and conversational metadata. By centralising these records within a shared data layer, DiNa supports continuity between remote monitoring, coaching, and clinician-facing review. Role-based access control and authenticated access mechanisms are used to protect privacy, maintain data integrity, and support compliance with relevant ethical and regulatory requirements.

The ingestion service manages monitoring data from mobile and wearable sources over two paths. Continuous background capture from battery- and connectivity-constrained devices, in particular smartwatches, is batched on the device and uploaded as files through pre-signed cloud upload URLs, stored under structured paths that preserve traceability and support de-identification. During an active coaching session, the same signals can be streamed to the orchestration service over a dedicated WebSocket channel (Section 3.6). The file path accommodates both raw sensor files, such as accelerometry, photoplethysmography, and temperature, and vendor-generated summaries such as heart rate, activity, and sleep metrics.

Asynchronous workers handle background processing tasks after uploading new data. These workers parse incoming files, transform them into structured records, and store the processed outputs for downstream use by the platform. They also support lifestyle-related processing, such as handling meal logs, including the identification of meal content and estimating caloric values using AI-assisted processing pipelines, as well as operational tasks such as delivering reminders and notifications. By separating these functions from the user-facing services, the platform supports background processing without placing the same load on interactive components.

Together, these backend services support the integration of sensing, conversational coaching, and clinician-facing coordination within a single platform architecture.

### 3.6 Wearable integration

The wearable integration was designed to support heterogeneous consumer health devices while maintaining interoperability and consistent data representation across the platform. The pipeline adopts the Open mHealth (OMH) standardisation framework (61), allowing data from different devices to be normalised into a common structure for longitudinal analysis and context-aware coaching.

Two integrations were implemented to demonstrate this capability: a Samsung Galaxy Watch 6 and a Dexcom continuous glucose monitor (CGM). Data availability varies by source. Core platform interactions, including conversation, logging, and goal actions, operate in real time, while wearable data may be streamed live, batch uploaded, or retrieved retrospectively depending on device and API constraints. These differences are handled explicitly by the platform and are described for each integration below.

#### 3.6.1 Samsung galaxy watch 6 integration

A custom smartwatch application was developed for continuous multisensor acquisition from the Samsung Galaxy Watch 6. The application captures heart rate (HR), photoplethysmography (PPG) at green, red, and infrared wavelengths, accelerometer data, skin temperature, and device status indicators including battery level, charger connectivity, and wearer detection.

The integration supports two acquisition modes. During background monitoring, sensor streams are buffered on the watch and uploaded as files through the ingestion service, reducing dependence on continuous connectivity and accommodating device battery constraints. During an active coaching session, the same signals are streamed live through a dedicated WebSocket channel. The orchestration service then makes the incoming physiological signals available to the coaching agent as part of the current interaction context.

#### 3.6.2 CGM

The platform also integrates Dexcom CGM data for diabetes-related coaching. This is the only sensor that currently has a regulatory imposed delay. Glucose measurements are retrieved through the Dexcom V3 API, which introduces an approximately three-hour vendor-imposed delay (62). Consequently, CGM data are used for retrospective analysis. Real-time glucose alerts, such as hypoglycaemia and hyperglycaemia warnings, are safety-critical functions provided by the regulated Dexcom application, and DiNa does not duplicate them. DiNa uses retrospective data to identify multi-day patterns, such as recurring post-prandial excursions or overnight variability, and frames coaching around these trends. Pattern recognition over days is insensitive to a delay of a few hours, and trend-level coaching aligns with the platform’s behavioural focus on sustained self-management habits over immediate glycaemic correction.

Glucose trends are presented alongside meal and medication records in both the mobile application and clinician dashboard. This supports the review of longitudinal glycaemic patterns, including reflection on responses to meals, medication, and other behaviours, and provides a basis for subsequent goal setting and coaching discussions. To make the data latency explicit, both interfaces display the timestamp of the most recent glucose reading.

## 4 Methods

We evaluated DiNa through a formative, task-based usability study designed to examine the interaction quality. Participants completed representative tasks spanning monitoring, logging, and conversational coaching while thinking aloud. The evaluation combined standardised measures, self-reported ratings, and observed interaction behaviour.

This formative evaluation examined the following research questions:

- **RQ1, usability and cognitive workload:** How do users perceive the usability and mental effort of interacting with a system that combines multiple CCM functions?
- **RQ2, task performance:** Can users complete workflows that span monitoring, logging, and coaching in a facilitated session, and what interaction breakdowns emerge?
- **RQ3, initial acceptability:** Do users perceive benefits in having monitoring, coaching, and tracking features within a single platform?
- **RQ4, interaction reliability:** Do users experience coherent transitions between functional components, and how are observed breakdowns distributed across tasks?

### 4.1 Study design

We conducted moderated, lab-based think-aloud sessions with 10 participants (5 women, 5 men; mean age=27.6 years, SD=3.75). Participants were recruited from the university community through a poster advertisement. Inclusion criteria were: (i) age 18 years or older, and (ii) regular use of at least one health or fitness tracking application. None of the participants reported a diagnosed chronic disease.

Young, healthy adults familiar with digital health tracking were recruited as surrogate users to identify interaction and pipeline issues before evaluation in a clinically representative population. This staged approach is consistent with formative HCI practice, in which small samples are used to identify high-impact usability issues before testing with target users (57). Clinical effectiveness, long-term engagement, and adoption among people with chronic conditions were outside the scope of this evaluation.

Participants varied in their baseline tracking habits. Although most (80%) did not regularly log meals, the majority reported tracking physical activity at least a few times per week. Commonly used tools included Apple Health, Samsung Health, Garmin, and spreadsheets. Participants reported a mean confidence score of 3.7 out of 5 for learning new health applications. Table 2 reports individual characteristics collected through a self-reported pre-session questionnaire, including age, self-described gender, meal-logging frequency, physical-activity tracking frequency, prior tool use, confidence in learning new health applications (5-point Likert-type scale, 1=not at all confident, 5=extremely confident), and chronic disease diagnosis status.

**Table 2:** Individual participant characteristics collected before the usability session.

ID	Age (years)	Gender	Meal logging	Activity tracking	Current tools/apps	Tech confidence (1–5)	Formal diagnosis
P01	27	Female	Daily	4–6 times/week	Apple Health	5	No
P02	26	Male	Never	Never	None reported	5	No
P03	20	Male	Never	Once/week	Samsung Health	3	No
P04	28	Female	Never	2–3 times/week	Apple Health; Spreadsheet/manual	4	No
P05	29	Male	Never	2–3 times/week	Apple Health	3	No
P06	30	Male	Daily	Daily	MyFitnessPal; Apple Health	3	No
P07	23	Female	Never	4–6 times/week	Heavy (other entry)	4	No
P08	30	Male	Never	Never	Apple Health	2	No
P09	31	Female	Never	Never	None reported	3	No
P10	32	Female	Never	Daily	Garmin	5	No

### 4.2 Procedure

Each session lasted approximately 45–60 min and followed a standardised protocol. After providing informed consent and completing a brief demographic questionnaire, participants were given a brief introduction to the application. Sessions were conducted on Android smartphones preloaded with DiNa .

The session consisted of two phases. In the first phase, participants freely explored the application while thinking aloud. In the second phase, each participant completed a series of structured tasks: (1) set a nutrition or activity goal, (2) log a recent meal (including photo capture), (3) review logged meal history, (4) interact with the digital coach to receive goal-related advice and (5) access quick health trends. These tasks were selected to span key components of the platform (monitoring, logging, and coaching), allowing us to observe how users transition between these functions within an integrated workflow.

The coaching task focused on the participant’s previously set goal; video-coded durations are reported in Table 3. The moderators observed the sessions and recorded screen activity, time on task, and any points of confusion, error, or breakdown. After completion of the tasks, the participants completed the usability and workload questionnaires, followed by a semi-structured exit interview exploring perceived value, pain points, and feature requests. All sessions were screen- and audio-recorded. During the Think-aloud, participants verbalised while exploring and performing each task (63). Moderators followed the interactive style common in usability practice (64): they prompted participants to keep talking, asked brief clarifying probes, and stepped in only when a participant could not proceed, noting each intervention. The audio was transcribed automatically with utterance-level timestamps. Because the recordings also captured the coach’s spoken output, the transcripts contain both the participants’ verbalisations and the coach’s delivered utterances. This study was approved by the Southern Health and Disability Ethics Committee, New Zealand (Ref: 2024 EXP 19184).

**Table 3:** Accounting of sample, questionnaire scores, observed breakdowns, and video-coded task performance (n=10).

Item	Value
Sample	n=10 (5 women, 5 men); mean age 27.6 years (SD 3.75); 0/10 reported a diagnosed chronic disease; mean tech confidence 3.7/5; 8/10 never meal-log
Session	Approximately 45–60 min laboratory visit, including exploration, five structured tasks, questionnaires, and interview
SUS	M=76.1 (SD 15.0); range 42.5–95.0
NASA-TLX	Overall M=26.7; mental demand 41.7; effort 32.7; performance 38.8; frustration 22.8; temporal demand 14.3; physical demand 9.9
Acceptability (5-point)	Likelihood of reuse M=3.4 (SD 1.17); recommendation M=3.8 (SD 1.32); perceived usefulness M=4.2 (SD 0.92)
Observed breakdowns	8/10 coaching tasks needed an unmute prompt or facilitator rescue; 4 participants encountered a logging mismatch (misclassification, portion error, or failed state update); 2 participants shifted away from manual meal entry
T1 Goal setting	Administered 9/10; completed 9/9; median 122 s (range 54–249)
T2 Meal log (photo)	Administered 10/10; completed 10/10; median 80 s (range 26–485)
T3 Review meal history	Administered 9/10; completed 9/9; median 25 s (range 3–81)
T4 Coach (goal advice)	Administered 10/10; completed 9/10 (one case required the facilitator to take over and was scored not completed); median 223 s among completed (range 72–591); unmute prompt or rescue in 8/10
T5 Health trends	Administered 7/10; completed 7/7; median 28 s (range 17–118)
Not administered	T1 (1), T3 (1), and T5 (3) were omitted when the session ran over its allotted time

### 4.3 Outcome measures

We collected both quantitative and qualitative data to evaluate the DiNa platform. Task performance was assessed using task completion rates, time on task, observed errors, and moderator intervention notes. Usability was measured using the System Usability Scale (SUS), while cognitive workload was captured using a short version of the NASA Task Load Index (NASA-TLX), selected to reduce participant burden in a formative usability session that already included multiple tasks, questionnaires, and an exit interview.

To assess initial acceptability, participants responded to three items on a 5-point Likert scale assessing their willingness to use DiNa again, recommend it to others, and perceived overall usefulness. Qualitative data from the interviews were analysed using reflexive thematic analysis following Braun and Clarke (65). Initial codes were generated by the first author and iteratively refined through discussion with a second author. Themes were developed inductively to capture recurring usability issues and interaction patterns.

Task completion and time-on-task for the five structured tasks were coded from the session screen recordings, from the start of the instructed task to save, on-screen success, or the moderator moving on. A task was scored completed if the success criterion was met, including with a moderator prompt; not completed if it was abandoned or required the moderator to take over; and not administered if that task was never given. Backend event instrumentation recorded timestamped goal creation, meal logging, tool calls, and conversational exchanges, and these logs were reviewed after each session to confirm that the events for each task had been captured. Latency and error-code summaries were not extracted from these logs for this study. As a single-session formative evaluation, this study does not assess long-term engagement or sustained use. It focuses on early interaction patterns and usability issues that may influence future adoption in longitudinal deployments.

### 4.4 Data analysis

SUS responses are scored following the standard procedure: odd items are scored as the response minus 1, even items as 5 minus the response, and the sum is multiplied by 2.5. With n=10, SUS is reported descriptively with a 95% confidence interval. Because SUS is a global rating of perceived usability, and task performance is recommended alongside it when evaluating eHealth technology (66), we complement SUS with observed think-aloud performance so that the questionnaire results and what participants actually did can be read together.

Think-aloud data are analysed in two passes. The first followed the reflexive thematic analysis. The second is a deductive coding pass in which the unit of analysis is the *integration touchpoint* rather than the individual feature: any moment in which a participant’s action, expectation, or comment spanned two or more components (monitoring, logging, goals and reminders, coach, review), or in which the output of one component was expected to surface in another. Each touchpoint is coded for its pathway, its outcome (coherent, self-recovered, moderator-recovered, unresolved breakdown, or voiced but not exercised), and any moderator intervention. The task-level results in Table 3 are retained as a feature-level reference.

To analyse BCT delivery, every coach turn captured in the transcripts is coded for the BCT Taxonomy v1 technique(s) it enacted (58), whether it referred to the participant’s own goals, logs, or statements, and for the participant’s immediate response (positive: accepts or elaborates; rejects or corrects; other). The immediate response indicates in-session acceptability and not behaviour change. They are applied to the full transcripts (recordings from the 10 participants).

## 5 Results

Participants completed the NASA-TLX, the 10-item SUS, and three acceptability items on a 5-point Likert scale after the usability session. Table 3 gives a compact accounting of the sample, questionnaire scores, video-coded task performance, and observed breakdowns, so that the questionnaire results below can be read against what participants actually did.

The NASA-TLX results (Figure 5) indicated a low to moderate overall cognitive workload, with a mean score of 26.7 across the six dimensions. Mental demand (M=41.7) and effort (M=32.7) were higher than the other subscales, whereas frustration (M=22.8), temporal demand (M=14.3), and physical demand (M=9.9) remained low. Perceived performance was rated at M=38.8. These scores describe perceived workload in a facilitated laboratory session and are interpreted alongside the breakdown counts in Table 3; they do not imply unassisted, error-free completion.

**Figure 5:**
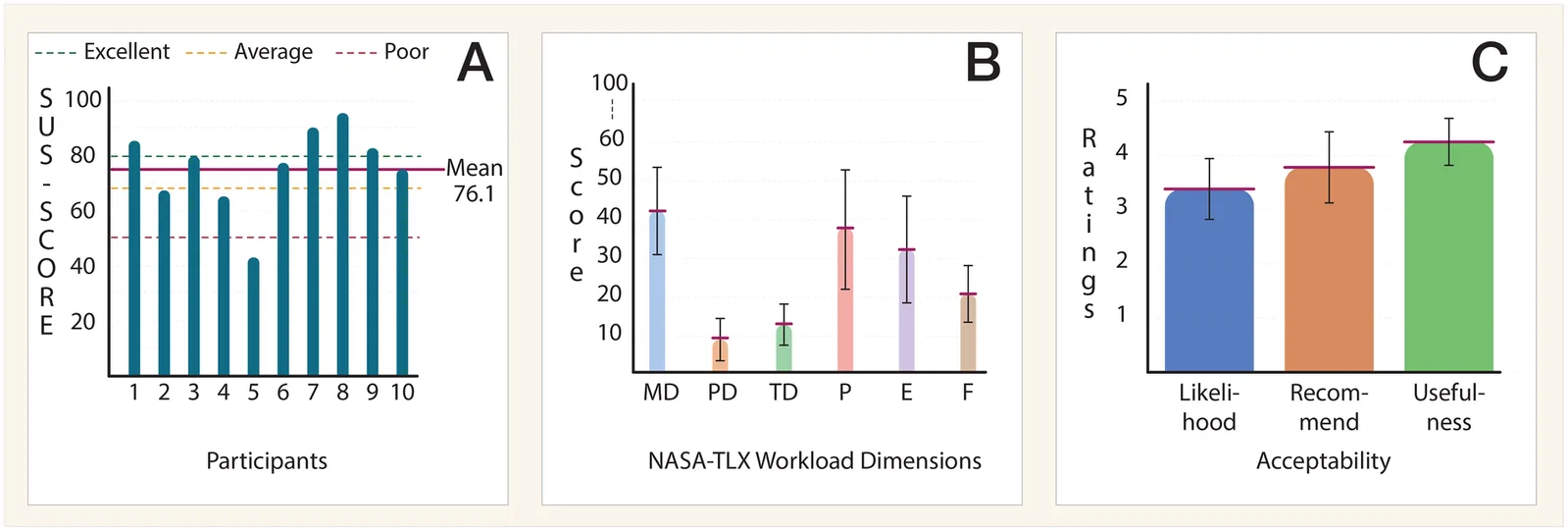
Usability, task load, and acceptability evaluation results. **(A)** System Usability Scale (SUS) scores for each participant (N=10). Dashed lines at 50, 68, and 80 indicate interpretability thresholds for poor, acceptable, and excellent usability, respectively. **(B)** NASA-TLX workload ratings averaged across participants for six subscales (MD: Mental Demand; PD: Physical Demand; TD: Temporal Demand; P: Performance; E: Effort; and F: Frustration). **(C)** Acceptability ratings for likelihood to reuse, intention to recommend, and perceived usefulness were measured on a 5-point Likert scale.

The mean SUS score was 76.1 (SD=15.0; 95% CI 65.4 to 86.8), with individual scores ranging from 42.5 to 95.0 (Figure 5). In this healthy-volunteer sample the mean falls in the generally good usability range, and most of the interval lies above the common benchmark of 68. Because the interval is wide at n=10, we interpret SUS together with what was observed in the sessions: the video-coded task performance (Table 3) and the think-aloud performance at integration touchpoints (Table 4). The same sessions included the microphone-mute and logging breakdowns quantified in Table 3, which shows that favourable questionnaire ratings coexisted with facilitator-assisted recovery in the coaching task.

**Table 4:** Think-aloud performance by integration pathway (10 participants; touchpoints with an observable outcome).

Where components meet	Touchpoints	Worked or recovered	Unresolved
Coach and goals or reminders (e.g., coach recalls or edits a goal)	27	27 (100%)	0
Coach and monitoring or review data (e.g., coach summarises the day)	18	15 (83%)	3
Logging and review (e.g., logged meal appears in the diary)	29	20 (69%)	9
Coach and logging (e.g., meal logged by talking to the coach)	48	33 (69%)	15
Goals and logging, monitoring, or review (e.g., goal progress in the diary)	30	14 (47%)	16
**Total**	**152**	**109 (72%)**	**43**

Worked or recovered: worked at first attempt or was recovered within the session by the participant or with a moderator prompt.

Bold values indicate totals across all integration pathways.

Acceptability ratings were more mixed. On the 5-point scale, participants reported a mean likelihood of regular use over the next month of 3.4 (SD=1.17), a mean likelihood of recommending DiNa to a peer of 3.8 (SD=1.32), and a mean perceived usefulness for supporting health and fitness goals of 4.2 (SD=0.92) (Figure 5). These ratings indicate moderate early acceptability, with perceived usefulness rated more positively than near-term use or recommendation. Together, these findings suggest a difference between questionnaire-rated usability and acceptability, with mixed near-term use intentions even among participants who completed the facilitated session.

Time-on-task in Table 3 is the video-clock interval from task start to completion or hand-off, not keystroke-level motor time.

### 5.1 Integration-level think-aloud performance

Think-aloud performance at the integration level followed the same trend as the SUS. Table 3 shows near-ceiling completion when features are assessed individually, and across the five pathways that connect the platform’s main components, most touchpoints worked on the first attempt or were recovered within the session, usually by the participants themselves (Table 4). Perceived usability and observed integration performance were therefore both generally good, supporting the feasibility of the integrated workflow. The coach-to-goals pathway worked throughout: the coach recalled and revised goals created in the goal screen, and participants verified the changes (“Oh, I can see them all here”). The coach also handled logging by conversation, and logged meals appeared in the diary and review screens in most cases. The unresolved cases, about a quarter of touchpoints, were concentrated in a few identifiable places that correspond to Themes 2 to 5 and indicate where refinement is needed. Goals did not yet appear in the diary or trend views (“I can’t easily find where my goals would be, but I can find my food”), and some actions reported by the coach, such as an edit or a deletion, were not reflected in the application (“Wait, it’s still there”). All participants logged a meal, and the coach drew on previously logged meals in 2 of 10 sessions. Because the task order placed meal review before the coaching task, the protocol did not exercise a complete chain from a logged meal and a goal, through coaching that used both, to review.

### 5.2 Delivered behaviour change techniques

We coded coach turns against the BCT Taxonomy v1 (58) (Figure 6). About half of the coach’s turns enacted a BCT, and about a third drew on the participant’s own data or statements. Self-regulation techniques were used most: self-monitoring through agent-mediated logging (2.3), goal setting (1.1), action planning (1.4), feedback (2.2) and goal review (1.5). Health information (5.1) and instruction (4.1) were the next most common. The participant received self-regulation techniques well and rarely rejected them. These techniques usually built on the participant’s own record. Information and instruction were received less well and accounted for half of all rejections, usually when the coach assumed a condition the participant did not have. Techniques grounded in the participant’s own data were accepted more readily than generic health content.

**Figure 6:**
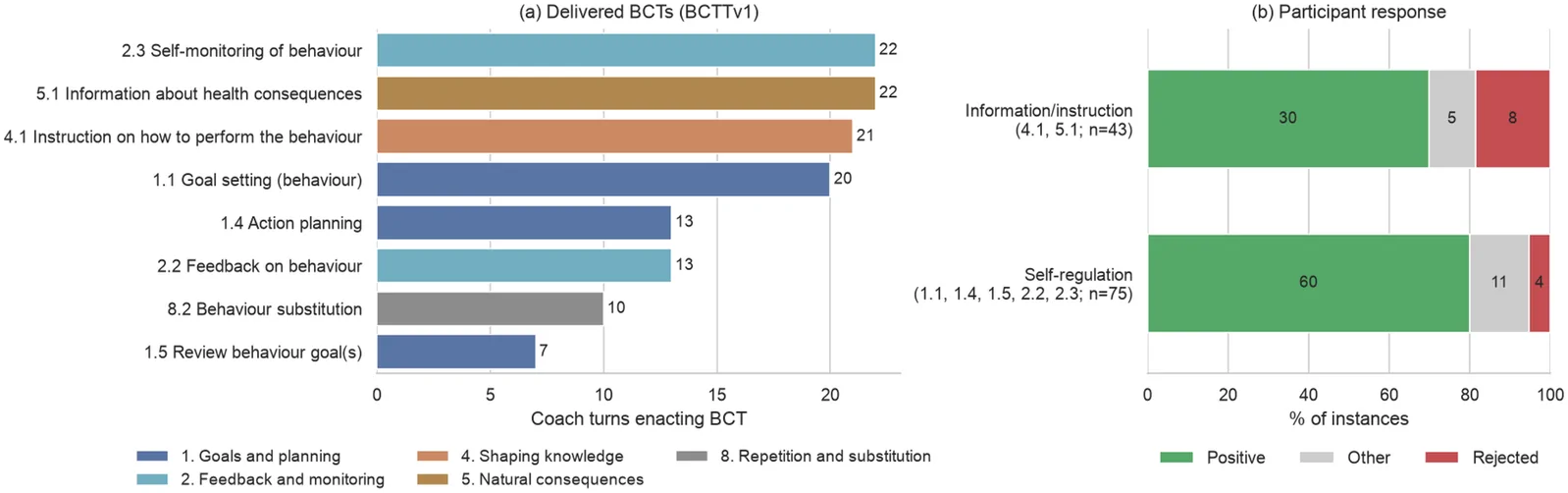
**(a)** Behaviour change techniques delivered by the coach (181 turns). **(b)** Participant response to self-regulation vs. information/instruction techniques; bar labels are counts.

### 5.3 Backend latency

Table 5 reports wall-clock minimum, maximum, and mean latency for the five application paths over five sequential single-user repeats. The four REST paths (goal creation, meal persistence, meal-history retrieval, and trend retrieval) returned HTTP 2xx on every repeat. Three of them had means between 34 ms and 47 ms. Meal persistence had a minimum of 42 ms, but a single 4.9 s response raised its mean to 1.0 s. Text coaching turns on the orchestration WebSocket took 3.6 s to 11.1 s (mean 6.5 s). Coaching-turn latency is therefore the dominant component of interactive response time on the platform, which is consistent with T4 having the longest time on-task in Table 3 and with participants’ uncertainty about whether the system was responding (Theme 4). During a separate, denser burst of requests from the same account, the authenticated GET /api/v1/auth/me endpoint returned HTTP 429. The API’s rate limiting engaged, as a condition a client must handle.

**Table 5:** Live single-user latency of the T1–T5 application paths.

Task	Path	HTTP	Min	Max	Mean
T1 Set goal	POST /api/v1/goals	201	30.4 ms	48.8 ms	37.0 ms
T2 Log meal (persist)	POST /api/v1/meals	201	42.2 ms	4906 ms	1017 ms
T3 Review history	GET /api/v1/meals	200	40.0 ms	58.5 ms	47.3 ms
T4 Text coaching turn	WebSocket conversationRequest → conversationResponse	5/5 replies	3.61 s	11.08 s	6.46 s
T5 Health trends	GET /api/v1/trends/me	200	26.7 ms	59.2 ms	34.1 ms

Five repeats spaced 2 s apart; values are wall-clock minimum, maximum, and mean. T2 is meal persistence, not photo analysis. T4 is text-chat coaching, not digital-human audiovisual delivery.

### 5.4 Qualitative findings

To complement the quantitative findings, we conducted a reflexive thematic analysis [Braun and Clarke (65)] of think-aloud transcripts and exit interviews. The initial codes were generated by the first author from the full corpus and then reviewed and refined with the second author through iterative comparison. Five themes were identified. These themes reflect not only local usability issues, but also interaction challenges that arise when monitoring, coaching, and tracking functionalities are combined within a single system. In particular, participants formed expectations about automation, personalisation, and workflow continuity when interacting with integrated features.

**Theme 1: High-frequency workflows amplified friction.** Photo-based meal logging was received positively, particularly because it reduced the need for active nutritional computation. P001 described the workflow as “really easy to just click and also take a picture and have all that information about the meal,” explaining that the automatic carbohydrate estimation removed the need to “count in my head,” and therefore made the feature suitable for everyday use. Similar reactions were observed across participants who preferred rapid-capture workflows over structured form entry. In contrast, manual logging was more frequently associated with interaction burden. Several participants paused or questioned the amount of required input during the workflow, and two participants explicitly shifted away from manual entry during the session. P002 reacted to the logging form with “Wow, that’s a lot of stuff to log,” later concluding, “I think I would be too lazy to log all these,” while P004 bypassed the form entirely by delegating meal logging to the conversational agent. Participants with prior self-tracking experience expressed a different concern: rather than reducing effort, they expected greater nutritional precision and control. P006, who reported regular calorie tracking outside the study, requested gram-level ingredient inputs and detailed calorie visibility, explaining that he wanted to “weight each of the ingredients and make sure that I am respecting this amount of calories.” These responses indicate that participants evaluated the same workflow through different expectations depending on their existing tracking practices and perceived need for precision.

Reliability issues further shaped how the workflow was interpreted. Four participants encountered situations in which logged outputs did not match their expectations, including food misclassification, incorrect portion estimation, and failed state updates. For example, P007 photographed a kiwi fruit that was recorded as a mandarin, P010 photographed a single slice of cake that appeared as two meals, and P002 observed that a duplicate meal entry remained visible after the system confirmed deletion. Participants did not treat these as isolated recognition errors alone, but as indications that the workflow could not always be trusted to maintain accurate system state. Participants also attempted to minimise the overhead of repeated interactions within these workflows. Rather than repeatedly re-entering meals, they expected rapid reuse and retrieval of prior entries. P008 requested quicker access to previously logged items so that “I can quickly find it again and add it,” reflecting an expectation that repeated actions should become progressively lower effort over time. These findings indicate that participants continuously weighed the effort of interactions against the perceived reliability and usefulness of the workflow.

**Theme 2: Goal setting lacked coherence with user expectations.** Participants’ difficulties with goal setting reflected a mismatch between how goals were represented in the system and how participants understood them in practice. Across participants, goals were described as flexible, contextual, and embedded in daily routines, whereas the system primarily supported predefined, fixed structures.

Several participants attempted to express temporal or situational variability in their goals, but found this difficult within the existing workflow. P001 wanted goals that reflect changing routines over days, comparing this to her own medication schedule: “my medication, I take one day, like, on even days I take a specific amount, but on the odd days I take another amount. So being able to select the days, for example, would be, like, quite great.” P002 similarly framed goals as time-bounded commitments that should later be reviewed in conversation with the system: “Maybe it would be good if I could set like a starting time and ending time. Like I want to commit to this for one month […] and then after a month […] I can talk to DiNa about how I did on this goal.” Participants also described goals that depended on multiple behavioural conditions or outcomes. P006, while setting a sleep-related goal, explained that he wanted to track not only sleep duration, but also sleep quality and routine: “I also want to have other criteria within that goal. For instance, going to bed at 10 pm and making sure I get enough REM and deep sleep.” These responses indicate that participants often understood goals as adaptive and context-dependent rather than as fixed daily targets.

Participants also encountered situations in which the system reinterpreted or constrained their intended behaviour. This was most visible during SMART goal generation. After P002 described exercise as a coping strategy for stress, the system reframed the goal as mindfulness practice, which P002 explicitly rejected: “But I don’t want mindfulness exercises. […] I just want to remind myself to go to the gym if I feel stressed because I know that I will feel better after going to the gym.” The reframing changed the intended meaning of the goal, leading to frustration and a reduced sense of control over the interaction. P005 described repeatedly attempting to activate the SMART goal function without receiving clear feedback: “there were like sometimes it says make it smart and I tried pressing that button and I didn’t get a response. So I wasn’t quite sure why I couldn’t do that.” Similar uncertainty emerged around action labels such as “Commit” and “Confirm,” which participants interpreted inconsistently or treated as redundant steps. In other cases, functionality existed but was not easily discoverable during goal creation, leading participants to rely on trial-and-error exploration or post-hoc editing.

Participants also frequently described goals in relation to other parts of the system, including coaching, reminders, and progress tracking, suggesting that goal setting was expected to function as an ongoing reference point across interactions rather than as an isolated configuration task. Despite these difficulties, participants consistently described goal setting as an important part of the system. P004 considered it “more important than logging a meal,” while others viewed goals as central to maintaining accountability, tracking progress, or guiding future behaviour. However, because goal-setting interactions were often experienced as rigid or unclear, participants sometimes treated the workflow more as a configuration task than as a flexible tool to manage daily behaviour.

**Theme 3: Coaching was accessible but not sufficiently personalised.**

Participants initially experienced DiNa’s conversational coaching as accessible and easy to engage with, particularly for lightweight tasks such as meal logging or basic queries. The conversational interface reduced interaction effort by allowing participants to bypass more structured input workflows. P001 valued being able to describe a meal conversationally when photo capture was missed, identifying “ the part where I describe my meal without even having to take a picture of it. If I forgot, for example, to take a picture before eating.” as a particularly useful affordance. P003 contrasted DiNa with conventional chatbots, describing her first interaction as “quite impressive based on […] her facial behavior and movements […] that was the thing that you can, you don’t usually see in other chatbots,” and adding that “it did it really well.” These responses suggest that participants experienced the agent as approachable and interactional lightweight during simple tasks.

However, once interactions moved beyond basic task completion, the participants began to evaluate the coaching in terms of contextual understanding and response quality. Several described the responses as repetitive or insufficiently adaptive. P006 reported that the agent often repeated the same explanation when asked for clarification: “if I ask to clarify things […] [it] just say[s] the same thing over and again.” He wanted responses to adapt to different communication needs, explaining that “people understand things differently.” During workload reflection, he also described becoming frustrated when responses felt repetitive or incomplete: “she wasn’t explaining things. She was repeating stuff or she just cut part way through.” Across participants, coaching was therefore evaluated not only by whether answers were provided, but by whether responses appeared responsive to the user’s specific query or communication needs.

Beyond informational responses, several participants expected the agent to take a more active guidance role. P004 described the coaching as overly affirming and insufficiently corrective: “I got, like, the impression, like, she’s always […] trying to say that you are right. And it’s never trying to contradict you.” She explained that she wanted clearer behavioural guidance: “if it’s a no, she should say no […] not in a rude, hard, or discouraging way, […] but kind of, you say no, and then you also explain why that is a no.” This suggests that participants expected the agent not only to provide information, but also to evaluate behaviours and justify recommendations. Participants also expected continuity across conversational interactions. P002 described attempting to retrieve information from an earlier interaction after the system had previously confirmed saving it: “I think there was a time, like we had a conversation and I ended it and then I started another conversation and tried to ask her if she remembered something from the previous,” referring specifically to a shopping list that could not later be retrieved. The failed retrieval created uncertainty about whether conversational outputs were being retained across interactions. These findings show that participants evaluated coaching not only in terms of conversational accessibility but also in relation to how well responses reflected prior interactions, personal context, and actionable guidance.

**Theme 4: Interface ambiguity disrupted the interaction flow.** Participants frequently encountered situations in which they could not clearly determine what the system was doing, whether actions had succeeded, or where information was located. Rather than remaining isolated usability issues, these ambiguities repeatedly interrupted task progression and shifted participants from goal-directed interaction to interpreting system behaviour. The most common form of ambiguity involved uncertainty about the system state during interaction. The clearest example was the muted microphone: 8 of the 10 participants did not initially realise it was muted at the start of the agent interaction, often requiring facilitator intervention before proceeding. Participants frequently paused mid-task to verify whether the system was actively listening or processing input. Both P002 and P008 asked aloud, “Am I muted?”, while P007 similarly questioned whether the system was responding at all: “I wonder if this is actually muted.” Participants also expressed uncertainty about whether voice recordings had been captured successfully or where recorded content was stored after interaction. In these situations, participants often stopped interacting with the task and instead attempted to diagnose the system’s current state.

A second form of ambiguity emerged around how actions and controls were interpreted across workflows. Several participants struggled to interpret labels, controls, or interaction outcomes consistently across workflows. The “Describe” button was repeatedly interpreted as unclear or ambiguous. When asked which feature she found most confusing, P009 identified it directly: “the only thing that I’m slightly struggling to get is this describe feature.” Similar uncertainty emerged during goal creation, particularly around action labels such as “Commit” and “Confirm.” P006 attempted to reason through their intended meaning during interaction, explaining: “commit is like, okay, are you sure about that goal? And then confirm is like, cool, your goal is set,” before concluding, “I’m not sure if you need two separate milestones for that.” Participants also hesitated around controls whose behaviour did not align with expectations, including multimodal features such as voice and video input, and in some cases, avoided interaction altogether when the expected outcome was unclear.

Participants additionally struggled to form a stable understanding of how information was organised across the system. Participants often expected information to appear in locations that did not align with the system’s structure. For example, P009 expected meal logs to appear within the Diary, while P006 wanted clearer separation between Goals and Diary, indicating uncertainty about where information should be created, stored, or retrieved. Similar confusion emerged when participants attempted to locate previously entered or generated content. These moments often disrupted transitions between features, as participants were required to search, infer, or test where information might appear before continuing the interaction. Presentation-level issues further disrupted interactions during conversational exchanges. Several participants reported difficulty following subtitles because they disappeared too quickly, overlapped with interface elements, or fell out of sync with spoken responses. P001 explained that “the subtitles should be there a little bit longer because it appears for like maybe five seconds […] And then the person doesn’t get to read through everything and it just disappears.” P003 similarly described being unable to fully read responses because subtitles were visually obstructed: “I couldn’t see exactly what was written because it was behind the relevant links and it disappeared quite fast.” P005 added that “the subtitles don’t quite sync,” making it difficult to verify spoken information in real time.

Across these situations, participants frequently shifted from smooth task progression toward trial-and-error exploration, interpretive pauses, or facilitator-supported recovery. In several cases, participants hesitated, avoided interaction, or relied on the facilitator’s guidance when the expected outcomes were unclear. Interface ambiguity was therefore experienced not simply as isolated confusion, but as a repeated disruption to the continuity of interaction across integrated workflows.

**Theme 5: Perceived value depended on integration quality.**

Across participants, DiNa was primarily evaluated in relation to existing self-management routines and tools rather than as a standalone application. Participants consistently recognised the value of bringing monitoring, logging, coaching, and review into a single platform, particularly compared with their current use of fragmented health applications and workflows. P001, described the appeal in terms of reducing tool fragmentation: “it’s really interesting to have everything in one app because, so for the case with one diabetic, you have a lot of different apps or things that you can use. But having everything in the same place is really interesting.” She linked this value directly to integration with tools she already relied on daily, explaining that “in my case, definitely [I would use it every day], because I would be able to connect my glucose sensor, because I also have the CGM, the Dexcom one,” and noting that photo-based logging could replace her current habit of mentally calculating carbohydrates for insulin dosing. Similarly, P002 imagined using the system to support communication with clinicians, explaining: “if my doctor asked me what I ate this week, I want to ask DiNa, can you just summarize it and then I can give it to the doctor.” These responses suggest that participants valued integration when it reduced fragmentation across existing health-management activities.

At the same time, participants did not evaluate integration as valuable in isolation. Instead, its usefulness depended on how reliably individual components functioned and connected within everyday workflows. Several participants explicitly compared DiNa to specialised tools they already used. P006 contrasted DiNa with his existing tracking ecosystem, explaining: “I use like MyFitnessPal to track my calories and that interface is quite nice for tracking calories […] And then I usually use Apple Health for other stuff.” When asked whether he would recommend DiNa to others already engaged in calorie tracking, he concluded: “I probably would recommend MyFitnessPal if I’ve been [tracking] calories before.” Rather than rejecting integration itself, participants evaluated whether integrated workflows could match or meaningfully extend the routines already established through existing applications. Participants who encountered issues with logging, goal setting, or coaching also frequently questioned the reliability of connected features such as summaries, recommendations, or future tracking, indicating that limitations in one component affected confidence in the broader workflow.

Perceived value also depended on participants’ existing engagement with self-management practices. Participants already involved in self-tracking or chronic condition management were more likely to identify situations in which integrated data and coaching could support everyday routines. For others, adoption remained conditional or situational. P009, who reported minimal personal engagement with health applications, explained that “I haven’t used any health apps […] at the moment I don’t think I will use this for my personal” use, but simultaneously recognised potential value for her father, who has diabetes, explaining that “he will definitely see with the meals how the glucose levels vary,” and concluding that the system “wouldn’t benefit someone like [me] with [my] preferences, but would benefit someone like [my] father who is diabetic.” These responses suggest that participants evaluated integration relative to their own health management needs, existing routines, and the perceived benefit of combining multiple functions within a single system. Overall, participants recognised the potential value of integration, but their confidence in that value depended on whether connected features could reliably support and align with existing self-management practices. Rather than evaluating DiNa solely as a collection of individual features, participants frequently assessed whether the integrated workflow could reduce effort, maintain continuity across interactions, and fit within the broader ecosystem of tools they already used.

**Outlier observations.** Individual differences in prior tracking practices and expectations shaped how participants interpreted and evaluated the system. P004 consistently responded positively to the integrated workflow and frequently compared outputs against her own estimates before accepting them. Reflecting on the agent-mediated meal logging, she explained: “I checked actually the carbs that I entered at noon and I was roughly okay. I counted the same things as her so it’s so good for me to check.” Despite encountering some of the same issues described in earlier themes, she remained willing to adopt the platform when outputs aligned sufficiently with her expectations. By contrast, P006 repeatedly questioned how outputs were generated and sought more detailed explanations for system behaviour. Discussing the photo-based nutritional estimates, he asked: “how does it, again, how does it know? Because people, if people were to use cheesecake, […] different recipes would have like slight variations in how much sugar they add, how much flour they add […] So like in that case, then how do you know these values?” He also suggested additional data integration for “diabetic management,” including blood pressure monitoring. Throughout the session, P006 frequently requested clarification about how values were calculated or how recommendations were derived. These contrasting cases indicate that participants interpreted the same integrated workflows differently depending on their prior experiences, expectations, and existing self-tracking practices.

## 6 Discussion

This study examined the usability and interaction characteristics of a prototype digital health platform designed to integrate functions relevant to chronic care, including monitoring, conversational coaching, and clinician-facing review. The findings provide early empirical insight into how such integration is experienced at the interaction level. In particular, the results highlight a distinction between (i) usability of individual features and (ii) perceived coherence and value of the overall workflow.

### 6.1 Feasibility vs. experience in integrated workflows

This study reveals a clear distinction between questionnaire-rated feasibility (RQ1, RQ2) and perceived value in use (RQ3). Participants worked through workflows spanning monitoring, logging, and coaching in a facilitated session, and rated the experience favourably on usability (SUS=76.1) and workload (NASA-TLX=26.7). Those ratings sit alongside the breakdown counts in Table 3, where 8 of 10 coaching tasks required an unmute prompt (7), facilitator rescue (1) and 4 participants encountered a logging mismatch, so they describe a facilitated session and not unassisted use. Within that scope, combining the three functions in a single interface did not produce prohibitive perceived workload in this healthy-volunteer sample, which is the feasibility result the study was designed to establish. It is not evidence of CCM usability in clinical populations. Questionnaire-rated feasibility also did not directly translate into a willingness to use the system in everyday contexts. Although participants described the system as “easy to use” and “straightforward,” their engagement was contingent on how it would perform over repeated use. Interaction effort was not treated as a static usability property, but as an ongoing trade-off against perceived benefit. This was evident in behavioural adaptations during interaction, where higher-effort actions such as manual logging were avoided or deferred (“Do I have to enter this manually?”), while lower-effort pathways such as photo logging or agent-mediated input were preferred, even when outputs were approximate.

This pattern aligns with prior work in personal informatics and digital health, which shows that sustained engagement is shaped less by initial usability and more by the ongoing cost–benefit balance of repeated interactions (67, 68). However, the present findings extend this work by showing how this dynamic operates in integrated systems, where effort is evaluated not only at the level of individual tasks but in relation to how those tasks contribute to downstream features such as coaching and summaries. In the integration-level analysis (Section 5.1), every participant logged a meal, yet the coach referred to previously logged meals in only 2 of 10 sessions. This was later identified as a software defect in how recent logs were supplied to the coach’s context, and not as a limitation of the design. Nevertheless, it illustrates the point: where logged meals did reach the coach, participants valued the result, and where they did not, the effort of logging was not visibly repaid downstream. Reliability in system state further shaped this experience (RQ4). Participants hesitated when they could not determine whether the system was listening, processing, or saving input, particularly during transitions across modalities such as voice, text, and navigation. While such issues are commonly discussed in terms of interaction breakdowns or execution gaps (69), in this context they did not stop the facilitated session, but they reduced confidence in how actions propagated through the system.

The conversational agent intensified this dynamic. While it reduced interaction effort and enabled flexible input, it also raised expectations that responses would reflect user data and prior interactions. When responses were perceived as repetitive or insufficiently contextualised (“she said the same thing no matter what I asked”), this reduced the perceived usefulness of the system as a whole. Prior work on conversational agents has highlighted their accessibility and engagement benefits, but also the importance of perceived relevance and grounding for sustained use (70–72). Our findings suggest that in integrated systems, these expectations extend beyond the agent itself to the system’s ability to utilise shared context. These findings establish a central paradox: users could use the system, but did not necessarily perceive it as valuable in its current form. This suggests that usability alone is insufficient to explain user experience in integrated CCM systems, and that value is determined by how well interactions support continuity, relevance, and reduced effort over time.

### 6.2 Integration and agent mediation shift evaluation to system-level coherence

A central finding of this study is that participants evaluated DiNa at the system-level rather than at the individual-feature level. Monitoring, logging, and coaching were not experienced as separate functionalities, but as a connected workflow, where outputs from one component were expected to inform and persist across others. Participants consistently treated outputs as shared context. Logged meals were expected to inform summaries and coaching; conversational interactions were expected to persist and remain accessible; and system actions were expected to produce visible and reliable state changes. When these expectations were not met, participants did not isolate the issue to a specific feature. Instead, they questioned whether the system as a whole was functioning as intended.

This finding extends prior work in digital health and CCM systems, which has largely evaluated monitoring, behavioural support, and care coordination as separate components (10, 11). While these studies demonstrate the effectiveness of individual functions, they do not account for how users interpret systems when these functions are combined. Our results suggest that integration changes the unit of evaluation: users shift from assessing whether individual features work to assessing whether the system behaves coherently across interactions. Our two levels of analysis illustrate this shift. Participants completed the individual tasks at or near ceiling. However, at the integration level, most touchpoints (72%) worked or were recovered, and the unresolved cases (28%) clustered where goals and coach actions connect to logging and review (Table 4).

This shift appears to be reinforced by the presence of the conversational agent. Rather than interacting with discrete tools, participants engaged with the system as a unified, responsive entity. Conversational input was treated as actionable intent, with the expectation that describing a behaviour (e.g., a meal or a goal) would result in corresponding system updates that persist across interactions. Prior HCI work has shown that conversational interfaces can blur boundaries between interface and system behaviour, leading users to attribute agency and coherence to the system as a whole (72). In the present study, this effect appears to amplify expectations of continuity and traceability across components. As a result, local inconsistencies were interpreted as system-level failures. Errors in food recognition, unclear totals, or failed updates were not treated as isolated inaccuracies but as signals of unreliable system behaviour. Similarly, when coaching responses did not reflect prior inputs or context, they were perceived as a lack of meaningful connection between components. Taken together, the thematic findings and the integration-level think-aloud results (Section 5.1) offer a new perspective on integrated CCM systems: integration, particularly when mediated by a conversational agent, shifts user evaluation from feature-level usability to system-level coherence, where continuity, traceability, and cross-feature reliability become primary determinants of perceived value.

### 6.3 Breakdown mechanisms in integrated systems

The qualitative findings identify three mechanisms that explain how breakdowns emerge within this system-level evaluation.

First, participants engaged in an ongoing effort–trust calibration, particularly in high-frequency tasks such as meal logging. Low-effort interactions (e.g., photo logging, conversational input) were accepted even when outputs were approximate, while higher-effort interactions increased expectations for precision and control. When these expectations were not met, participants questioned the system’s reliability more broadly. This aligns with prior work on the burden of self-tracking and the effort required to sustain personal informatics practices (67), but extends it by showing how this calibration operates across interconnected features, where outputs are reused for coaching and feedback.

Second, breakdowns emerged from mismatches between the system’s structure and users’ mental models. Goal-setting interactions illustrate this clearly. Participants attempted to define goals in ways that reflected real-life routines (e.g., temporal flexibility, multi-criteria goals), but encountered rigid structures that did not accommodate this complexity. Such mismatches have been widely documented in HCI as a source of interaction difficulty (69), and in behaviour change research as a barrier to effective goal setting (58). In the present context, however, these mismatches reduced not only the usability of individual features but also their perceived role within the broader workflow.

Third, participants expected continuity across interactions, particularly regarding conversational outputs and system state. When outputs were not retrievable or when system changes were not visibly reflected, participants expressed uncertainty about whether actions had occurred. This reflects broader findings in human–AI interaction, where trust depends on the system’s ability to maintain and communicate state over time (71, 73). In an integrated system, such breakdowns disrupted the perception of a persistent, stateful system and affected confidence in subsequent interactions. Importantly, these mechanisms did not operate independently. Effort, mental models, and continuity interacted to shape how users interpreted system behaviour. While prior work has identified these factors within individual systems, our findings show how they combine and propagate across components in integrated platforms. In this context, breakdowns are not isolated usability issues, but mechanisms through which local inconsistencies influence system-level evaluation.

### 6.4 Behaviour change techniques in an integrated platform

In this context, the techniques that worked best were the self-regulation techniques that the coach could enact on the participant’s own record, namely goal setting, action planning, goal review, self-monitoring, and feedback on behaviour, whereas generic health information attracted half of the rejections (Section 5.1). This agrees with meta-analytic evidence that self-monitoring combined with other self-regulation techniques is associated with larger effects (74), and that goal setting and problem solving are the techniques most used in health coaching for type 2 diabetes, where the number of techniques is unrelated to effectiveness (75). Integration matters here because goal review and feedback require the coach to hold the user’s goals and logs, which DiNa provides through its shared data layer and action tools. In the sessions, this loop, from self-monitoring to feedback against the goal to an action plan written back to the platform, ran end to end in the goal-centred conversations, but for logged meals, it was interrupted by the defect noted in Section 6.1, and elsewhere the coach fell back on generic education. The priority for the next version is therefore to have the coach draw on the user’s record consistently. These observations describe in-session acceptability among healthy volunteers, not behaviour change. We will report whether the coaching improves adherence and glycaemic control in our registered pilot trial (ANZCTR ACTRN12626000280303).

### 6.5 Comparison with prior work

Existing CCM platforms have largely been developed and evaluated around individual components, such as monitoring (e.g., MySugr), behavioural coaching (e.g., Noom), or care coordination (e.g., Epic MyChart), with relatively few systems integrating all three. Comparisons with these platforms are therefore positional and not benchmark-equivalent. The DiNa values reported here were obtained from a younger volunteer sample that was not recruited as a diabetes cohort, whereas the comparator platforms have been evaluated in clinically representative populations and, in several cases, under different evaluation paradigms.

As shown in Table 6, prior work demonstrated the effectiveness of these components in isolation, including improved self-monitoring, behaviour change, and clinician communication (10, 11). Comparatively less attention has been paid to how these components interact when combined within a single system. These findings extend prior work on chronic care management platforms, which has often evaluated monitoring, behavioural support, or care coordination as separate functions. Our results suggest that integration introduces a different interaction challenge: users form expectations not only about whether individual features work, but about whether data, coaching, and review operate coherently across features. In this sense, integration offers a functional advantage through shared-context reuse, but also introduces a system-level vulnerability whereby breakdowns in one component can reduce trust in the overall workflow.

**Table 6:** Comparison of representative chronic care management platforms across the three pillars of patient monitoring, patient activation, and care coordination.

Platform	Patient monitoring	Patient activation	Care coordination	Main limitations
MySugr	Glucose tracking and manual self-logging	Limited behavioural support features	Minimal provider integration	Focused primarily on monitoring; lacks integrated coaching and broader care coordination
Noom	Limited physiological monitoring	Behavioural coaching and habit-change support	No integrated clinical coordination	Strong emphasis on activation, but limited biosignal integration and clinician linkage
Epic MyChart	EHR-linked data and some connected device inputs	Limited behaviour change support	Provider–patient communication and dashboard-based coordination	Strong in coordination, but limited real-time monitoring and patient activation features
SmartHeart	Wireless physiological sensor integration	Rule-based voice coaching via Alexa	Clinician dashboard with alerts	Prototype focused on heart failure; coaching is rule-based and condition-specific
DiNa	OMH-normalised wearable ingestion (Galaxy Watch 6 with in-session streaming and background file upload; Dexcom CGM with ∼3 h vendor delay) and active self-logging	LLM-powered embodied conversational coaching with tool-based actions and a closed MI/BCT/CBT technique vocabulary (fidelity not yet coded)	Clinician dashboard with biosignals, logs, goals, and retrospective review (clinician evaluation pending)	Formative laboratory evaluation with n=10 healthy volunteers; wearable path not exercised in the usability study; two vendor integrations to date; clinical-population and longitudinal evaluation pending

This interpretation aligns with prior usability evidence from monitoring-focused systems. Reported SUS values for MySugr (approximately 55 and 70 across different evaluations) reflect a single-pillar app focused primarily on monitoring (76). In this context, DiNa’s mean SUS of 76.1 suggests that adding coaching and coordination layers does not necessarily reduce usability relative to monitoring-only designs. However, the comparison should be interpreted cautiously because the study populations differed: MySugr evaluations were conducted in clinically representative diabetes cohorts, whereas the present formative study involved younger surrogate users without diagnosed chronic conditions. Accordingly, part of the observed SUS difference may be attributable to sample characteristics rather than platform design quality alone. A related comparison concerns activation-focused systems such as Noom. Published evaluations of Noom primarily report behavioural and clinical outcomes (e.g., weight and glycaemic endpoints) and engagement measures, rather than standardised interaction-level usability metrics such as SUS (11). Accordingly, the Noom evidence base is strongest for intervention effectiveness, whereas DiNa contributes complementary evidence on interaction-level usability in an integrated platform that combines activation, monitoring, and coordination.

Similarly, Epic MyChart is best considered a functional and architectural comparator rather than a direct usability benchmark. Published MyChart reports primarily emphasise portal use, patient-clinician communication, and adoption-oriented workflow outcomes, rather than controlled task-level SUS assessments as used in the present study (12). Direct SUS comparisons with DiNa are therefore methodologically non-equivalent at this stage. Compared with single-pillar systems, this integrated architecture offers a specific functional difference: the same user-generated event can be reused across pillars without manual transfer (e.g., a meal log can support self-tracking, trigger coaching context, and remain available for coordinator review). In monitoring-only or coaching-only products, these processes are often separated across tools, which reduces contextual continuity and increases the burden of interpretation for users and care teams.

At the same time, integration changes failure characteristics. In more modular, single-function workflows, a local issue may remain visible only locally. In integrated workflows, local failures (such as logging ambiguity or generic coaching output) can propagate to perceived system-level quality. This dual pattern of contextual advantage coupled with failure risk is a key qualitative insight of the present study.

### 6.6 Design implications

Across these findings, design implications converge on a common requirement: in integrated, agent-mediated systems, individual features cannot be designed or evaluated in isolation, as users interpret system behaviour at the level of workflow coherence and continuity.

- **Treat high-frequency workflows as trust-critical infrastructure.** Repeated interactions, such as the meal logging, function as anchors for system credibility. In this study, participants tolerated approximation when interaction effort was low, but expected higher accuracy and control when effort increased. Errors or friction in these workflows were not contained locally; they reduced confidence in downstream features such as coaching and summaries. Designing for these workflows therefore requires not only rapid capture (e.g., photo or conversational input), but also visible correction and confirmation mechanisms that make system state and data quality explicit. For example, systems could support inline correction immediately after capture (e.g., editable recognition outputs with portion controls) or provide confidence indicators that signal uncertainty in automated entries. These mechanisms allow users to quickly verify and adjust data before it propagates to summaries and coaching. Evaluation should extend beyond task completion to include error recovery, correction latency, and the persistence of inaccuracies across connected features.
- **Align goal representations with how users structure behaviour over time.** Participants attempted to define goals in relation to real-life routines (e.g., temporal variation, multi-criteria targets), but encountered rigid structures that did not support this flexibility. This mismatch reduced the perceived usefulness of goal-setting and weakened its connection to tracking and coaching. Design should therefore support goals as evolving, context-dependent constructs rather than static entries, with explicit links to ongoing behaviour and system feedback. In integrated systems, goals must remain interpretable across components, enabling meaningful referencing in monitoring and conversational interaction. In this study goals were interpretable to the coach, which recalled and revised them, but not to the rest of the application: the pathway from goals to logging, monitoring, and review had the most unresolved touchpoints (16 of 30, affecting 8 of 10 participants). Concretely, this could include goal templates with adjustable parameters (e.g., weekday vs weekend targets), hierarchical goal structures (e.g., sleep quality composed of duration and deep sleep), and timeline-based views that allow users to track progress across days. Such representations enable goals to remain interpretable and usable across tracking dashboards and conversational interactions.
- **Make the grounding of conversational responses perceptible within interaction.** Participants evaluated coaching not only on accessibility, but also on whether responses reflected their data and prior interactions. When this grounding was not evident, responses were interpreted as generic, reducing the perceived value of the system as a whole. Rather than increasing system complexity, design should focus on making the basis of responses legible within the interaction (e.g., referencing recent logs or active goals). In integrated systems, perceived personalisation depends as much on visible linkage to user data as on underlying model capability. For instance, responses could explicitly reference recent user inputs (e.g., “based on your lunch today…”), active goals, or recent trends, and provide lightweight summaries of relevant data within the dialogue. This makes the linkage between data and advice visible without requiring full transparency of underlying models.
- **Design interaction states as first-class elements in integrated workflows.** Participants frequently hesitated when the system state was unclear (e.g., whether the system was listening, processing, or saving input), particularly during transitions between modalities. These moments introduced interpretation effort and disrupted interaction flow. In integrated systems, such ambiguities affect not only individual tasks but also the perceived coherence of the workflow. Design should therefore make the interaction state explicit and persistent across modalities, helping users understand how actions are processed and how information moves between components. Examples include persistent indicators of system state (e.g., active listening, processing, saved), explicit confirmation of completed actions, and recoverable interaction flows when users enter incorrect modes. In multimodal systems, transitions (e.g., from voice to logging) should preserve context and make the outcome of user actions immediately visible.
- **Evaluate systems at the level of workflows, not isolated features.** Participants consistently assessed the system based on how its components worked together, rather than as independent features. Breakdowns in one component affected perceptions of others, indicating that evaluation at the feature level is insufficient for integrated systems. Future studies should directly assess chained workflows connecting logging, goals, coaching, and review within a single interaction sequence, which the present feature-based protocol did not (Section 7). Given the use of a digitally literate proxy cohort in this study, subsequent evaluation should also examine how these workflows perform in clinically relevant populations with differing self-management demands. This may involve scenario-based evaluations that require users to complete end-to-end tasks (e.g., log a meal, set a goal, receive coaching, and review outcomes), as well as metrics that capture cross-feature effects, such as error propagation, breakdown recovery, and consistency of system state across interactions.

Together, these implications identify concrete design and evaluation priorities for researchers and practitioners developing integrated CCM systems.

### 6.7 Contributions

This work contributes initial design and formative usability insights into integrated CCM systems at both the conceptual and the system level.

First, we provide empirical evidence that integration changes the way users experience and evaluate digital health systems. Rather than simply combining functionalities, integration raises expectations for automation, personalisation, and workflow continuity, and shifts user evaluation from individual features to the coherence of interactions across components. Whether these patterns generalise to clinically representative chronic care populations remains to be tested.

Second, we present a modular CCM platform architecture that links patient monitoring, conversational coaching, and clinician-facing review through shared data flows. The system enables the reuse of patient-generated data in self-management, coaching, and retrospective analysis, supported by standards-orientated data handling (e.g., OMH, FHIR-compatible interfaces) and a unified backend that connects sensing, interaction, and review.

Third, we introduce an embodied conversational agent that integrates dialogue with tool-based system actions, such as logging, goal management, and data queries, and we specify its model, retrieval corpus, memory, and guardrail design in enough detail for replication. While this design enables flexible and accessible interaction, our findings highlight the importance of grounding responses in user data and maintaining continuity across interactions to meet expectations in integrated settings.

Finally, we implemented a shared data layer and backend event instrumentation that record goal creation, meal logging, tool calls, and conversational exchanges as auditable events. Clinician-facing workflows were not evaluated in this study, and the instrumentation was used to confirm that events were captured for each task, not to derive latency or error statistics. It provides the foundation for the clinician-dashboard review task and the reliability reporting planned for the next evaluation.

## 7 Limitations and future work

This study has limitations related to the study design, sample, system maturity, and scope of evaluation.

First, the evaluation was a laboratory-based, single-session usability study with n=10. This design is appropriate for identifying interaction and workflow issues in an early-stage system, and it did so, but the findings should not be read as evidence of real-world adoption, sustained engagement, or clinical effectiveness. The single-session design also cannot capture longitudinal use, where digital health interventions often experience high early attrition (38). Longitudinal deployment is therefore the next evaluation stage.

Second, the sample consisted of university-recruited, relatively young, digitally literate volunteers (mean age=27.6 years) without diagnosed chronic disease. As set out in Section 4, this was the first stage of a staged evaluation, chosen to keep clinical risk low, to simplify ethics for an unvalidated system, and to isolate pipeline and usability defects from the additional demands of the target population. Transfer to that population will not be automatic. Older adults, people with multimorbidity, lower health or digital literacy, polypharmacy, incomplete sensor adherence, and clinician workflow constraints introduce interaction and self-care barriers that were absent in this cohort (77). We therefore expect SUS to fall below, and NASA-TLX to rise above, the values observed here. Hyzy et al. report a digital-health SUS benchmark near 68 after excluding physical-activity apps (78), so the present mean of 76.1 should be read as an upper reference from a favourable sample and not as an estimate of CCM usability. The next study will recruit adults with at least one chronic condition across a range of ages and health-literacy levels, retain SUS, NASA-TLX, task completion, and time-on-task for comparability, and add wearable sync reliability and a clinician-dashboard review task.

Third, the system is an early-stage implementation with technical constraints that are documented in Sections 3.3, and 3.6 and summarised here. In-app conversation, logging, goal actions, and in-session watch streaming are interactive over WebSocket transport. Dexcom glucose data are delayed by approximately three hours under vendor API policy and are used retrospectively with timestamps shown. The coaching agent’s guardrails were not clinically safety-evaluated, and no fidelity coding of the delivered MI, BCT, or CBT techniques was performed. The backend is designed for horizontal scaling and has a recorded failure-mode register, but it has been exercised only in ten laboratory sessions, and no throughput, tail-latency, or load-test figures exist. Backend event instrumentation was reviewed to confirm event capture and was not used to derive latency or error summaries. The next deployment phase will instrument turn latency, wearable sync, and tool-call error rates, run load testing against the failure-mode register, and code technique fidelity on a sample of transcripts.

Fourth, the evaluation focused on patient-facing interactions with the smartphone application. Clinician-facing dashboard workflows were not assessed with clinicians, and the clinician review task noted above is planned as next step.

Finally, production failure behaviour remains unexamined. Since ten laboratory sessions cannot speak to clinic-scale behaviour, API outages, or concurrent multi-user load. These limitations reflect the early stage of the platform and define the longitudinal, clinical, and system-level evaluations needed to assess the benefits and challenges of integration in CCM systems.

## 8 Conclusion

This work described the design of the DiNa platform and reported a formative usability evaluation of its integrated interaction model. The platform brings together patient monitoring, conversational coaching, and clinician-facing review within a single modular architecture, with the coaching agent, retrieval corpus, guardrails, and wearable pathways specified in detail. In a controlled usability study with healthy volunteers (n=10), questionnaire scores indicated generally good usability (mean SUS 76.1) and low to moderate cognitive workload (mean NASA-TLX 26.7), and video-coded task performance quantified the breakdowns that accompanied those scores. Analysed by integration pathway, most cross-component touchpoints worked or were recovered within the session, with unresolved breakdowns concentrated where goals and coach actions connect to logging and review. Self-regulation techniques grounded in participants’ own data were received more positively than generic health information.

The findings also indicate that integration alone is not sufficient. Logging workflows, goal-setting interactions, coaching specificity, and device interoperability remain important constraints on the overall user experience, and these should be addressed before broader deployment or clinical evaluation.

In summary, these findings provide early evidence of interaction feasibility in healthy volunteers and support integrated CCM systems as a promising design direction to reduce fragmentation across monitoring, behavioural support, and retrospective review. They should not be interpreted as evidence of usability or acceptability in clinically representative chronic-care populations. Future work will prioritise refinement of the identified workflow issues, clinician-centred evaluation, and longitudinal testing with clinically representative samples.

## Data Availability

The datasets presented in this article are not readily available because sharing of participant recordings and transcripts is restricted under the conditions of ethics approval. Requests to access the datasets should be directed to the corresponding author.

## Footnotes

^1^https://www.soulmachines.com/.
