# Supplementary material for: Design and formative usability evaluation of a prototype integrated digital health platform for chronic care support
Source: Front Digit Health. 2026 Oct 22;8:1780895. doi: 10.3389/fdgth.2026.1780895 (PMC13735224; doi:10.3389/fdgth.2026.1780895)
Supplement: Supplementary file 1 [file Datasheet1.pdf]

Supplementary Material

1 DETAILED PLATFORM COMPONENTS

1.1 ECA Design

Within the application, the embodied conversational agent (ECA) is a core interaction component. The ECA is designed around two main elements (Figure S1): a dialogue manager and a coaching agent. The dialogue manager maintains the structure and state of the coaching session, helping the conversation remain aligned with the current task and supporting session resumption after interruptions.

The coaching agent interprets user requests and can invoke a set of tools to perform specific actions. These include retrieval-augmented question answering from a curated health knowledge base, logging of meals and medications, goal creation and updates, reminder management, note-taking, and access to historical data. This tool-based design allows the ECA to combine dialogue with concrete platform actions rather than operating only as a text generator.

The dialogue manager governs the conversational structure and flow of the interaction, ensuring that the conversation remains coherent with the coaching session. A coaching session refers to a goal-oriented interaction between the user and the ECA designed to support self-management of health through education, reflection, and behaviour change. These sessions are typically similar to the themed real-world coaching sessions (e.g., medication adherence, physical activity planning) and can consist of subtasks such as data review, personalised feedback, and goal setting or action planning.

When a user interacts, the conversation flows through the dialogue manager and maintains session-level interactions and conversational tasks accordingly. These dynamics enable efficient continuous conversation management and work better with LLMs to adhere to the task instructions without diverging into unrelated

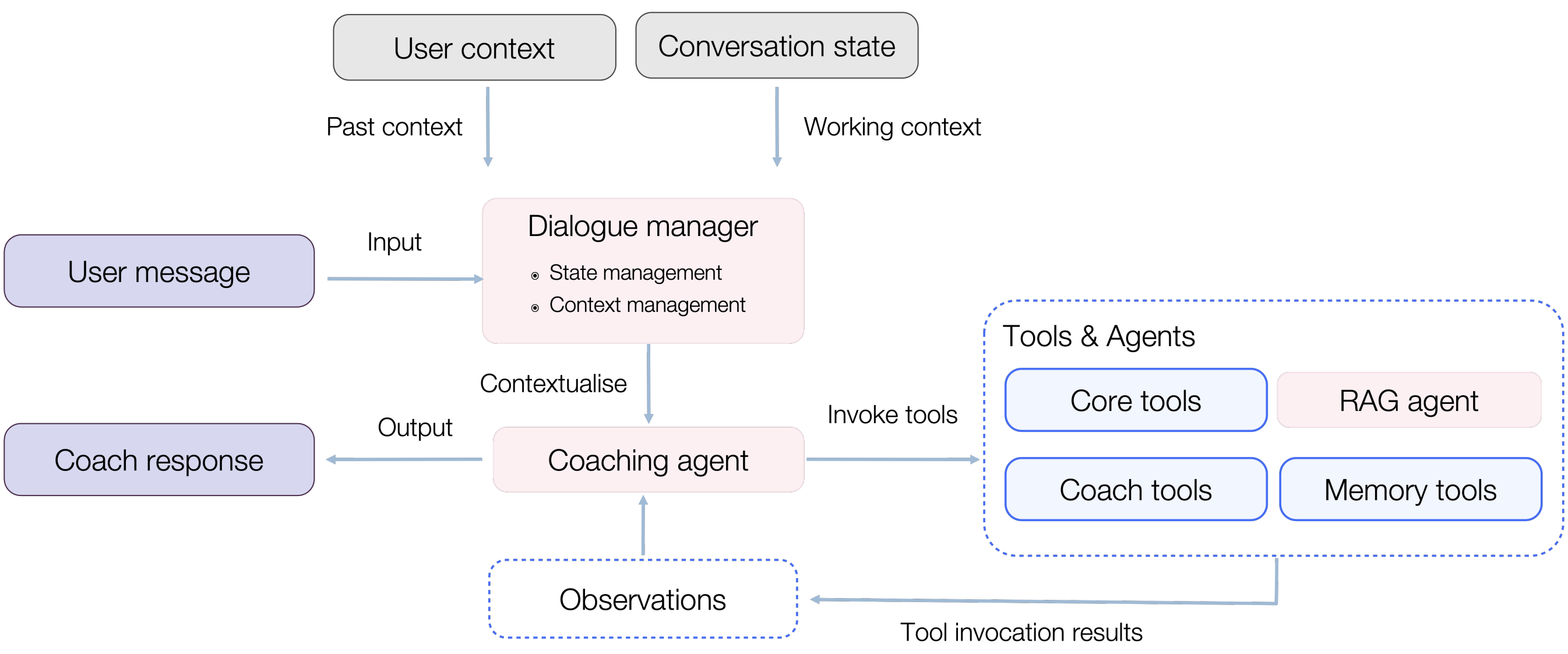

Figure S1. Overview of the conversational flow and modular structure of the ECA. User queries are processed through a dialogue manager that maintains context and session flow, followed by a coaching agent that dynamically invokes specialised tools for reasoning, data retrieval, and personalised feedback generation, enabling adaptive and context-aware digital health coaching.

topics. The dialogue manager persists the state of the conversation flow and provides capabilities to resume the conversation from where it left off. It ensures that the ECA can handle interruptions and session resumptions seamlessly, providing a consistent user experience across multiple interactions.

The coaching agent is the core of the ECA, responsible for understanding user inputs and executing tasks. It is designed as a multi-step tool calling agent, capable of invoking various tools to perform specific actions based on user requests. Every tool call from the agent captures explicit reasoning in a parameter, ensuring a reduction in hallucination risks and increasing interpretability. This ensures that actions of the ECA are reliably grounded in the user context and allows debugging and improving the prompts.

The coaching agent has common built-in tools for essential tasks, including:

- **Messaging Primitives**: `send_msg_to_user` for final responses and `send_ack_message` to acknowledge work in progress while tools run.
- **Knowledge Agent**: The AI agent integrates a specialised knowledge agent that employs retrieval-augmented generation (RAG) techniques with a validated health knowledge base to provide evidence-based information. This custom tool enables contextually grounded and accurate responses to patient health, lifestyle, and CCM questions, ensuring that therapeutic recommendations align with current medical guidelines and best practices.
- **Health Data Tracking and Logging**: Comprehensive logging and tracking of self-reported patient data, including meals (`log_meals`), medications (`log_meds`), symptoms, and biomarkers, supporting personalised adherence strategies and maintaining robust patient engagement logs. The system includes data retrieval capabilities to access historical health records and generate trend analyses.
- **Goal Management**: Tools such as `set_goals`, `update_goals`, `delete_goals` allow the AI to collaboratively define, edit, and monitor patient health goals and corresponding action steps.
- **Reminder Management**: The agent incorporates reminder functions, automatically managing personalised health-related notifications, such as medication schedules, goal checks, and activity reminders.
- **Notes Management**: The agent can create and update notes such as grocery lists, recipes, education bites, etc.

The coaching agent also integrates comprehensive therapeutic strategies through the coaching response strategy tool, which dynamically loads behaviour change techniques (BCT), motivational interviewing (MI) and cognitive behavioural therapy (CBT) approaches based on patient context and therapeutic objectives. The system supports essential MI techniques alongside core CBT interventions delivering BCTs for health behaviour change. It allows the research team to debug and improve the prompts used for generating responses, ensuring that the agent interactions are grounded in evidence based practices and tailored to individual patient needs.

The system provides a standardised interface for custom tool definition, allowing developers to seamlessly integrate specialised capabilities while maintaining consistent interaction patterns and error handling protocols. This approach facilitates the integration of specialised agents such as a knowledge agent for evidence-based health information retrieval, data tracking tools to capture self-reported patient metrics, and data retrieval utilities to access historical health records and trends. Each custom tool inherits the built-in validation, error handling and self-healing capabilities of the system, ensuring robust operation and consistent user experience across all functionalities.

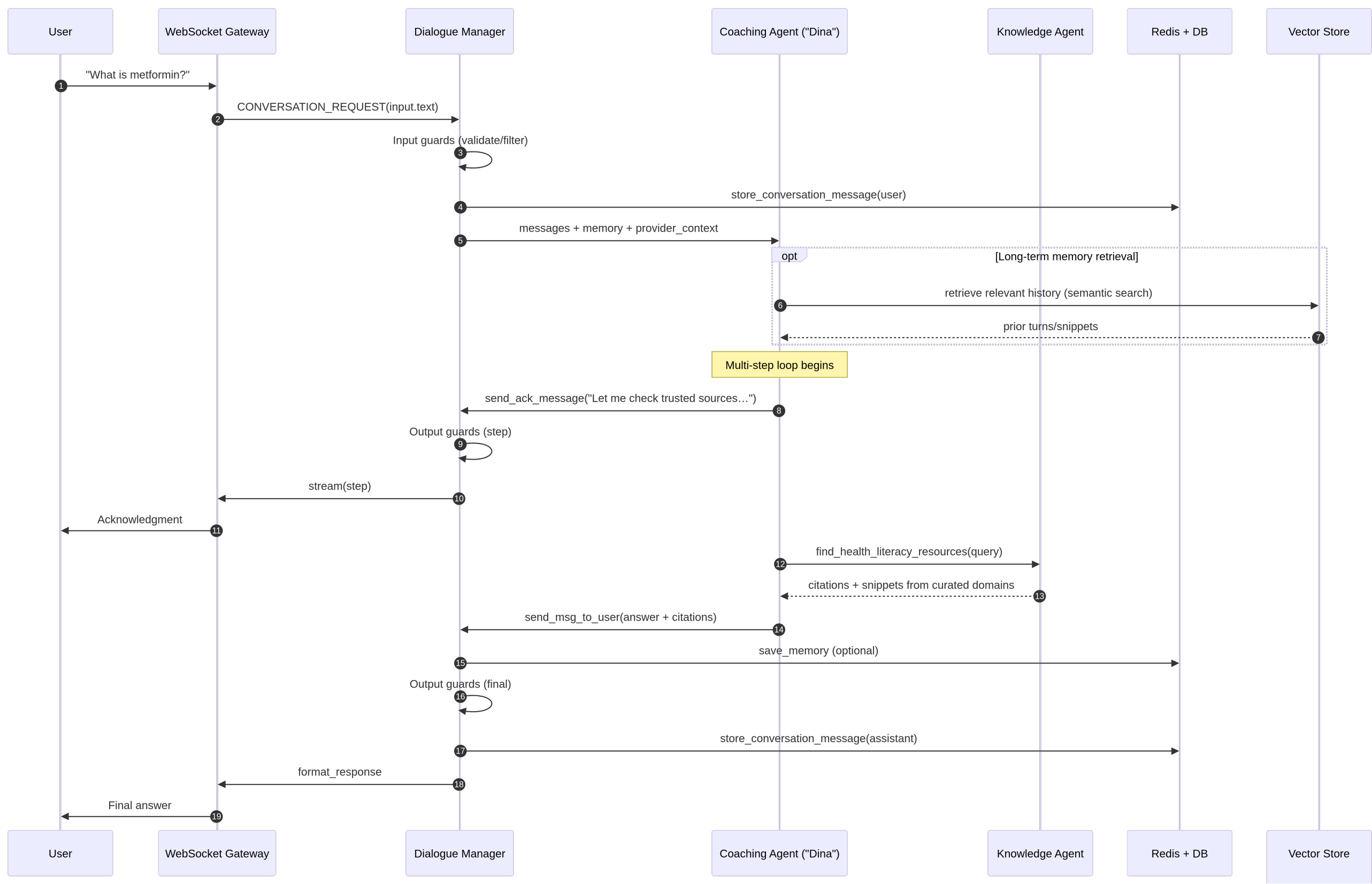

Figure S2. The sequence of how a user's health literacy question (e.g., "What is metformin?") flows through the system from input to cited answer

The same integration pattern used for the knowledge agent applies to other specialised agents. Any capability with a clear input–output contract can be wrapped as a tool with a JSON schema and a handler, inheriting common logging, safety checks, and error recovery without changes to the dialogue manager. Examples include care navigation and appointment booking, symptom triage and data science agents, data connectors (e.g., wearables or EHR summaries), or programme-specific goal planning. This enables incremental expansion of functionality while keeping the coaching loop stable.

The coaching agent is also enriched with the context of the conversation, including user preferences, historical interactions through memory, and individual-level data such as sleep patterns, activity metrics, self-reported meals, and medication data on demand through tools. This context-aware design allows the agent to provide personalised responses and maintain continuity throughout sessions, improving user engagement and satisfaction. This ECA employs three primary memory manipulation tools: `memory_insert` and `memory_replace` to actively update and refine its episodic memory of past experiences and procedural memory of personalities and response patterns, and `recall_memory` to access previous sessions, thus providing effective and long-term conversational memory. Conversational histories are embedded into vectors and stored in a vector database, allowing the agent to retrieve relevant past interactions and maintain context throughout sessions.

The current conversation context is used as working memory, which is dynamically updated as the conversation progresses. This combination of short-term memory for adaptability and long-term continuity significantly enhances conversational coherence and accuracy over prolonged usage periods. Conversation history can also be compacted through summarisation so the coach retains salient context without exceeding

model limits. ECA further includes a `todo_write_tool`, where agent can create, update and mark complete the tasks without relying solely on conversation history. This allows the ECA to manage long-horizon tasks effectively even after context has been compacted.

As the coaching agent runs in a continuous loop, it has an inherent self-healing mechanism, which operates as part of the core agent execution cycle. This allows continuous result evaluation and error recovery through a structured validation that evaluates each tool execution outcome against expected parameters and contextual appropriateness. Specifically, the correction loop is composed as: (i) *result validation*, where each tool execution output is assessed for completeness, accuracy, and contextual relevance using predefined validation criteria and semantic coherence checks; (2) *error detection and classification*, which identifies potential misunderstandings, incomplete executions, or contextual misalignments through pattern matching and logical consistency verification; and (iii) *adaptive recovery*, where the decision agent automatically initiates corrective actions, including parameter adjustment, alternative tool selection, or conversation repair strategies.

This self-correction capability significantly improves interaction robustness by reducing conversation breakdowns, minimising user frustration from agent errors, and maintaining a therapeutic relationship through seamless error recovery. The system maintains an internal count mechanism that triggers escalation protocols when repeated correction attempts fail, ensuring graceful degradation and appropriate hand-off procedures when automated recovery is insufficient.

To ensure patient safety and appropriate ethical interactions, the agent includes integration for guardrails explicitly prompted to prevent the generation or delivery of harmful or inappropriate content.

This comprehensive agent architecture, supported by LLM capabilities, ensures effective, personalised, and context-aware communication. It acts as an intelligent digital health coach and assistant capable of actively supporting CCM and sustained patient engagement with the platform.

1.2 Backend System

CCM platforms must support diverse user needs, high data volumes, and evolving feature sets without compromising reliability or responsiveness. To meet these requirements, the backend of was designed as a modular service architecture (Figure S3) that supports conversational coaching, multimodal data ingestion, and access to patient data across patient- and clinician-facing interfaces.

As shown in Figure S3, the backend comprises four main functional components: an orchestration service, a data service, an ingestion service, and asynchronous worker services. Together, these components manage real-time interactions, persistent storage, cloud-based uploads, background processing, and synchronisation across the mobile application, conversational agent, and coordinator dashboard.

1.2.1 Orchestration Service

The orchestration service coordinates interactions between the mobile application, the embodied conversational agent powered by an LLM, and the platform data layer. The agent can be delivered through a digital avatar, voice interface, or chat interface. During each session, the orchestration service retrieves user context, including physiological measurements, medical background, lifestyle logs, and prior interactions, to support context-aware coaching.

Session histories are retained to preserve continuity across repeated engagements and to support later analysis of user engagement and conversational patterns. To support low-latency interactions, the service

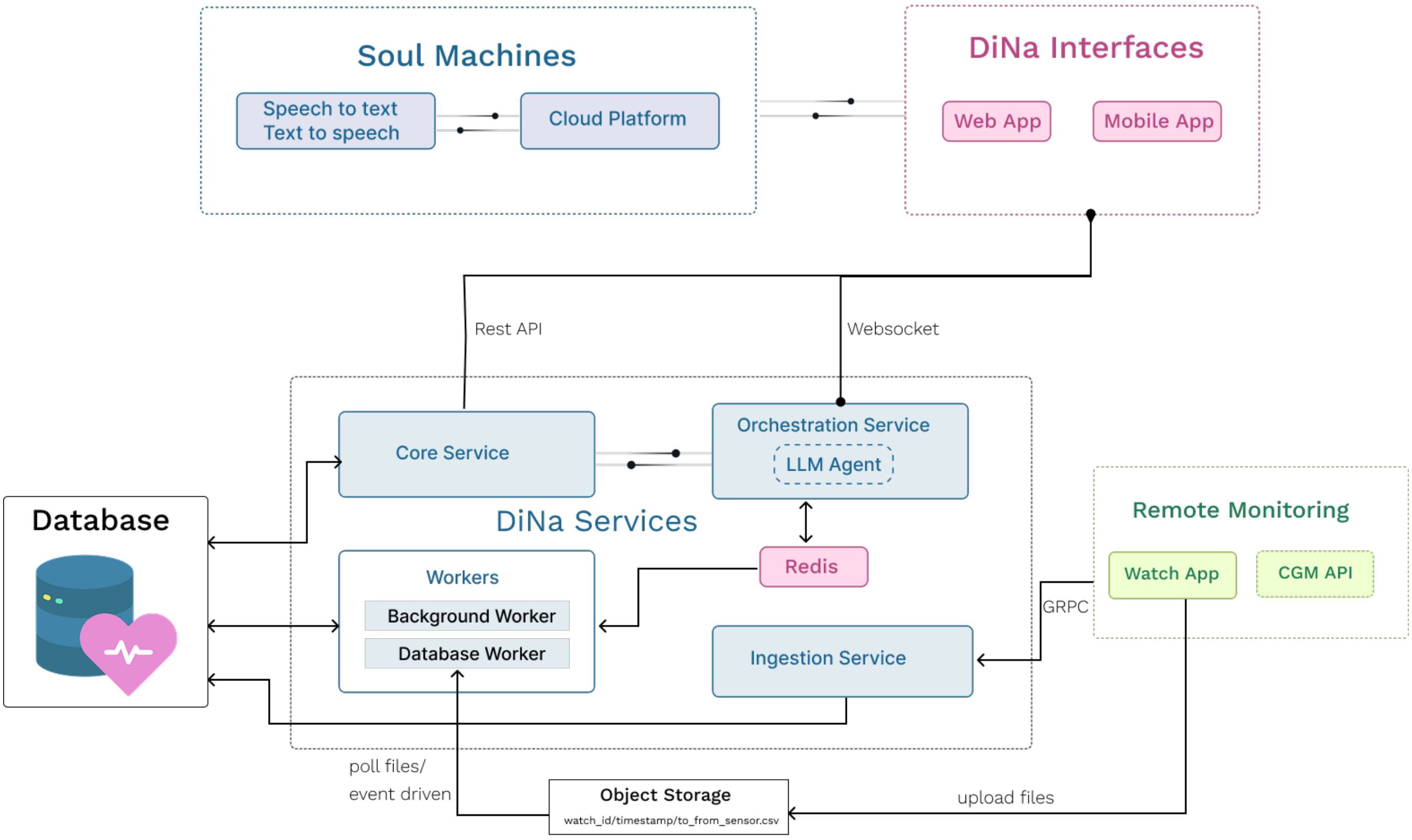

Figure S3. Backend architecture of the platform. The orchestration service coordinates real-time LLM interactions across interfaces, while the ingestion and worker services manage biosensor data uploads, processing. Redis supports caching and WebSocket scaling, ensuring responsive, continuous synchronisation between remote monitoring and coaching.

uses WebSocket-based communication, while Redis supports caching, publish-subscribe messaging, and synchronisation across concurrent sessions.

1.2.2 Data Service

The data service provides the persistent data layer for the platform. It maintains patient records, device registrations, biosignal data, lifestyle logs, action plans, treatment-related information, and conversational metadata. By centralising these records within a shared data layer, supports continuity between remote monitoring, coaching, and patient-facing care coordination.

Role-based access control and authenticated access mechanisms are used to protect privacy, maintain data integrity, and support compliance with relevant ethical and regulatory requirements.

1.2.3 Ingestion Service

The ingestion service manages high-volume data uploads from mobile and wearable sources. Because some devices, particularly smartwatches, are battery- and connectivity-constrained, the platform primarily uses file-based upload workflows, while supporting real-time ingestion when appropriate.

Uploaded data are transferred using pre-signed cloud upload URLs and stored using structured paths (`sensorid/timestamp/filename`) to preserve traceability and support de-identification. This ingestion pipeline accommodates both raw sensor streams (e.g., accelerometry, photoplethysmography, temperature) and vendor-generated summaries (e.g., heart rate, activity, and sleep metrics).

1.2.4 Workers

Workers are asynchronous backend microservices that process data and perform background tasks independently of user-facing services. These workers parse incoming files, transform them into structured records, and store processed outputs for downstream use by the platform.

They also support lifestyle-related processing, including meal log handling with meal-content identification and caloric estimation using AI-assisted processing pipelines **?**, as well as operational tasks such as delivering reminders and notifications. By separating these functions from interactive services, the platform supports responsive user interactions while sustaining background throughput.

Together, the backend services support integration of sensing, conversational coaching, and clinician-facing coordination within a unified platform architecture.
